# The discovery of a key prenyltransferase gene assisted by a chromosome-level *Epimedium pubescens* genome

**DOI:** 10.3389/fpls.2022.1034943

**Published:** 2022-11-14

**Authors:** Guoan Shen, Yanjiao Luo, Yu Yao, Guoqing Meng, Yixin Zhang, Yuanyue Wang, Chaoqun Xu, Xiang Liu, Cheng Zhang, Gang Ding, Yongzhen Pang, Hui Zhang, Baolin Guo

**Affiliations:** ^1^ Key Laboratory of Bioactive Substances and Resources Utilization of Chinese Herbal Medicines, Ministry of Education, Institute of Medicinal Plant Development, Chinese Academy of Medical Science & Peking Union Medical College, Beijing, China; ^2^ Institute of Animal Sciences, The Chinese Academy of Agricultural Sciences, Beijing, China; ^3^ Key Laboratory of Plant Molecular Physiology, Institute of Botany, Chinese Academy of Sciences, Beijing, China; ^4^ College of Life Sciences, University of Chinese Academy of Sciences, Beijing, China; ^5^ Chongqing Key Laboratory of Traditional Chinese Medicine Resource, Chongqing Academy of Chinese Materia Medica, Chongqing, China; ^6^ Key Laboratory of Biodiversity Science and Ecological Engineering, Ministry of Education, College of Life Sciences, Beijing Normal University, Beijing, China

**Keywords:** *Epimedium pubescens*, genome assembly, prenyltransferase, prenylated flavonoids, whole genome duplication

## Abstract

*Epimedium pubescens* is a species of the family Berberidaceae in the basal eudicot lineage, and a main plant source for the traditional Chinese medicine “Herba Epimedii”. The current study achieved a chromosome-level genome assembly of *E. pubescens* with the genome size of 3.34 Gb, and the genome guided discovery of a key prenyltransferase (PT) in *E. pubescens*. Our comparative genomic analyses confirmed the absence of Whole Genome Triplication (WGT-γ) event shared in core eudicots and further revealed the occurrence of an ancient Whole Genome Duplication (WGD) event approximately between 66 and 81 Million Years Ago (MYA). In addition, whole genome search approach was successfully applied to identify 19 potential flavonoid PT genes and an important flavonoid PT (*EpPT8*) was proven to be an enzyme for the biosynthesis of medicinal compounds, icaritin and its derivatives in *E. pubescens*. Therefore, our results not only provide a good reference genome to conduct further molecular biological studies in *Epimedium* genus, but also give important clues for synthetic biology and industrial production of related prenylated flavonoids in future.

## Introduction

**Figure 1:**
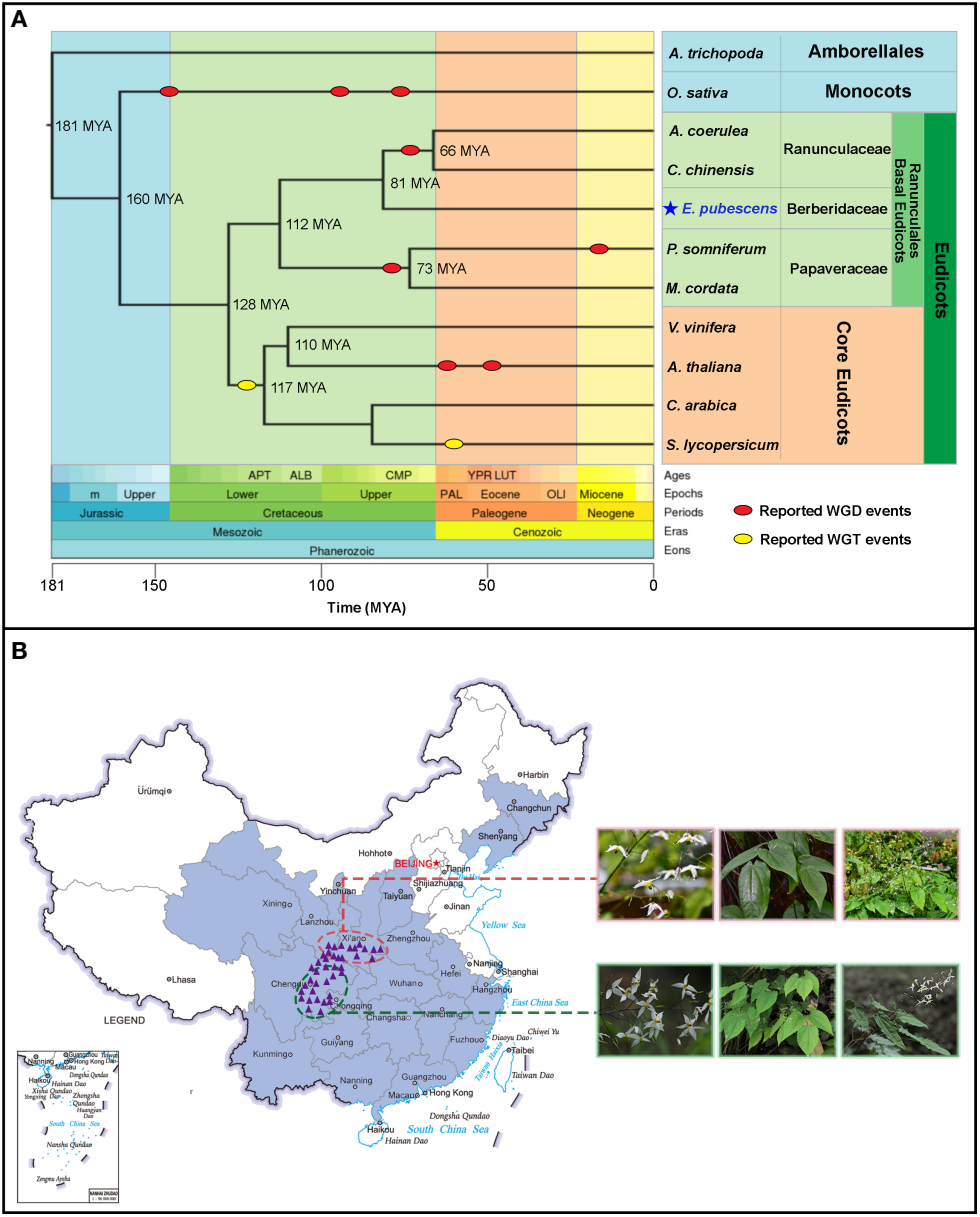
The phyologentic position of *E*. *pubescens* from Timetree website and the geographical distributions of *Epimedium* and *E*. *pubescens* in China. **(A)** Timetree of selected taxonomic groups in angiosperm. Whole Genome Duplication (WGD) events (red oval) and Whole Genome Triplication (WGT-γ) events (yellow oval) were shown on the Timetree; the divergence times among different taxonomic groups on the phylogenetic tree were predicted by Timetree online services (http://www.timetree.org/); hierarchy classifications of taxonomic groups was highlighted with different colors on the right of Timetree, based on Angiosperm Phylogeny Group IV (APG IV). **(B)** Geographical distributions of *Epimedium* and *E*. *pubescens* in China. The purple line delimited the border of China, the light blue line delimited the border of Chinese coast. The geographical distributed area of *Epimeidum* was highlighted in cyan color on the map of China; the distribution areas of *E*. *pubescens* were indicated with purple triangles and split into two groups circled with red and green dash lines respectively (the geographical locations were provided in Table S2 and sampling details were described in **Supplemental Information**). Two *E*. *pubescens* groups possessed different morphological features, including flowers, young leaves, and whole plants.

*Epimedium* L., the largest herbaceous genus within the family Berberidaceae, order Ranunculales according to the updated Angiosperm Phylogeny Group IV (APG IV) system (**Figure 1A**) (Byng et al., 2016), contains more than 60 species occurring unevenly from North Africa (Algeria) to East Asia (Stearn et al., 2002). With more than 50 species identified, China is believed to be the recent diversity center of *Epimedium* genus (De Smet et al., 2012). Herba Epimedii is totally made of leaves from *Epimedium* plants and well-known as “Yinyanghuo” in the traditional Chinese medicine for more than 2000 years (Chinese Pharmacopoeia Commission, 2020). Besides the extraordinary pharmaceutical activities for treating sexual dysfunction, *Epimedium* plants could confer other benefits to human health, including anti-tumor, anti-antiosteoporosis, cardiovascular protective, and neuroprotective effects (Zheng et al., 2014; Rouger et al., 2016; Wang et al., 2019). It has been verified by modern pharmacological studies that 8-prenylated flavonol and its glycosides are the bioactive components in *Epimedium* plants (Ma et al., 2011; Ming et al., 2013; Mbachu et al., 2020).

*Epimedium* belongs to basal eudicots and is a key sister group to core eudicots (Byng et al., 2016). There exists an ancestral Whole Genome Triplication gamma (WGT-γ) event (Soltis and Soltis, 2016; Clark and Donoghue, 2018) before the divergence of core eudicots 128 Million Years Ago (MYA) (**Figure 1A**) from basal eudicots. Moreover, a lack of such an ancestral WGT-γ event has been revealed in multiple species from Ranunculales of basal eudicots (Jaillon et al., 2007; Filiault et al., 2018; Guo et al., 2018; Liu et al., 2021c). In addition, Whole Genome Duplication (WGD) events are believed to widely appear in eudicot species during their genome evolution (Sankoff and Zheng, 2018) (**Figure 1A**). Such events are critical in shaping the genome structure among different plants (Wu et al., 2020). However, it is so far less known about the whole genome replication events in *Epimedium* genome evolutionary history.

In China, *Epimedium* species are unevenly distributed across temperate mountain regions (**Figure 1B**). Among which, wild populations of *E. pubescens* with a broad morphological diversity are widely distributed at altitudes from 300 to 2000 meters in Southwest of China (**Figure 1B**) (Gao et al., 2011; He, 2014; Liu et al., 2017a). *E*. *pubescens* is an important species of *Epimedium* with high and stable level of 8-prenylated flavonoids as a major commercial source for Herba Epimedii (Chinese Pharmacopoeia Commission, 2020). In addition, *E. pubescens* as a diploid species (2n=2x=12), possesses the chromosomal structure of karyotype symmetry type of 2A, which is previously reported to be similar to most *Epimedium* species (**Table S1**) (Zhang et al., 2018; Wang et al., 2020). Therefore, *E. pubescens* is a suitable representative species for constructing the reference genome of *Epimedium* for answering evolutionary questions and the exploring biosynthetic mechanisms of medicinal compounds in Herba Epimedii.

In this study, a chromosome-level genome assembly was achieved for *E. pubescens* and the further analyses confirmed that *E. pubescens* genome did not experience the WGT-γ event shared by core eudicots, but possessed an ancient WGD event during its evolutionary process. A genome guided *PT* gene search revealed 19 potential prenyltransferases (*PT*s) in *E. pubescens*, and especially *E. pubescens PT8* (*EpPT8*) were further confirmed as an enzyme for the production of 8-prenylated flavonols. The reference genome of *E. pubescens* provided new insights into the early evolution events of this species, and could potentially facilitate the design of breeding strategy in improving the germplasm resources of Herba Epimedii.

## Materials and methods

### Plant materials

*E. pubescens* plants were grown in the germplasm nursery at the Shawan District (29°N, 103°E), Leshan city, Sichuan province of China. In the germplasm nurseries, *Epimedium* plants were grown and covered with a black shade net to avoid direct sun exposure. Wild populations of *E. pubescens* were thoroughly investigated during field trips from 2018 to 2019 by Dr. Chaoqun Xu (**Table S2**) and totally 39 samples of *E. pubescens* were identified by Professor Baolin Guo. Voucher specimens from these wild populations were deposited at the herbarium of the Institute of Medicinal Plant Development (IMPLAD), Beijing, China, under the voucher numbers from B. L. Guo00841 to B. L. Guo00879. Fully developed leaves were detached in the spring of 2018 and washed with double-distilled sterile water, flash frozen in liquid nitrogen and stored at -80°C until DNA extraction and sequencing. Fresh roots, shoots, leaves, flowers, and fruits from the same individual plant were harvested and immediately frozen in liquid nitrogen, and stored at -80°C for RNA extraction and RNA sequencing.

### Genomic DNA sequencing and chromosome-level genome assembly

Genomic DNA was extracted from the leaves using the CTAB DNA extraction protocol (Varma et al., 2007). DNA concentration and purity were examined using NanoDrop and Qubit (Thermo Fisher Scientific, MA, USA), and DNA integrity was assessed using the pulsed-field electrophoresis. The genomic DNA was then used to construct libraries with an average insert size of 300 bp, and sequenced on the Illumina Novaseq 6000 platform (Illumina, San Diego, CA, USA) with average sequencing depth at about 230.94 × (771 Gb). For ONT sequencing, the high-quality genomic DNA was separated using the BluePippin™ System (Sage Science, USA), and purified to construct a library with size ranging from 15 to 50  kb using ONT template prep kit (SQK-LSK109, Oxford headquarters, USA) and NEB Next FFPE DNA Repair Mix kit (New England BioLabs, MA, USA). The high-quality library was sequenced on the ONT PromethION platform with MinION flow cell (R9.4.1) and ONT sequencing kit (Oxford headquarters, USA. Finally, a total of 906 Gb raw data (271.32 ×) was generated with an average ONT length of 19.79 kb and an N50 of 31.17 kb (**Table S3**, **S4**). Raw data was processed for base calling by Oxford Nanopore base caller using default parameters (Wick et al., 2019), and ONT reads were trimmed with Porechop (https://github.com/xxz19900/Porechop). Finally, these reads were assembled into contigs using SMARTdenovo (Liu et al., 2021a). The assembled contigs were then polished with Illumina short reads three times by Racon (Vaser et al., 2017) and Pilon v1.20 (Walker et al., 2014). Finally, BWA-MEM2 (https://github.com/bwa-mem2/bwa-mem2) for alignment of short reads and BUSCO 4.0.6 with viridiplantae_odb10 (Simao et al., 2015) were used to assess the quality and completeness of the assembly.

### Genome annotation and transcriptomic analysis

For the prediction of gene models, *ab initio* prediction program Augustus v2.4 and evidence/homology-based strategies were applied to annotate genomic contigs and transcriptomic data and the results were integrated into final gene models using EVM v1.1.1 (Haas et al., 2008). For repeat annotation, the sequences of genome assembly were subjected to structural and *ab initio* prediction of repeats using LTR_FINDER v4.0.6, RepeatScout v1.0.5 and RepeatMasker v4.0.6. For non-coding RNA annotation, microRNA, rRNA, tRNA, and other functional RNA were predicted by combining several strategies. For functional annotation, the predicted gene models were subjected to homology searches against the following databases: NCBI non-redundant protein sequences (NR), Kyoto Encyclopedia of Genes and Genomes (KEGG), Clusters of Orthologous Groups of proteins (KOG/COG/eggNOG), Gene Ontology (GO), Pfam and Swiss-Prot/TrEMBL (**Table S5**). In the RNA-seq analysis, raw data was cleaned by Trimmomatic software v0.39 and the gene expression levels were determined with Hisat2 and Stringtie. The differentially expressed genes were detected by R package DESeq v1.10.1. Co-expressed gene network was inferred from the FPKM values in different tissues (FPKM > 10) using weighted gene co-expression network analysis (WGCNA) (Langfelder and Horvath, 2008).

### Comparative genomic analyses

Protein sequences from *E. pubescens* and other 11 angiosperms (**Table S6**) were clustered into orthologous groups using Orthofinder software v2.3.3 and aligned by MUSCLE v3.8.155147. The phylogenetic tree was built using the maximum-likelihood method with 1,000 bootstrap replicates in RAxML v8.2.1248. Synonymous substitution rate per synonymous site (Ks) was calculated by MCScanX software. Ks peaks were determined by using the Genome_tools (Ks_Density_plot.r, https://github.com/ZhangXu-CAS/Genome_tools/), and the Ks distribution plots were made using R package ggplot2. The times of Ks peaks were calculated by the formula, Ks/13×1000 (MYA) (Gaut et al., 1996). The divergence times among different taxonomic groups were estimated by the TimeTree online service (Kumar et al., 2017).

### Phylogenetic analysis of candidate prenyltransferases

To identify the candidate prenyltransferase genes, protein sequences from the known plant flavonoid prenyltransferases were used to search the *E. pubescens* genome using BLASTP with E-value of 10^−5^. The candidate sequences were further submitted to NCBI CDD database to confirm the presence of conservative domains. The protein properties of putative EpPTs, including physical and chemical properties, subcellular localization, transmembrane (TM) *α*-helices and the presence of chloroplast transit peptides (cTP) were predicted using the online ExPASY ProtParam tool (http://web.expasy.org/protparam/), TargetP online server (http://www.cbs.dtu.dk/services/TargetP/), TMHMM 2.0 (http://www.cbs.dtu.dk/services/TMHMM/), ChloroP 1.1 (http://www.cbs.dtu.dk/services/ChloroP/) and PSORT (http://psort1.hgc.jp/form.html), respectively. The sequence alignments were generated using ClustalW (Li, 2003), and the gene tree was constructed using RAxML package v 8.13 with 1,000 bootstrap replicates (Stamatakis, 2014). The best substitution model was determined using BestModel.

### Cloning and *in vitro* functional characterization of prenyltransferase genes

Total RNA from fresh leaves of *E. pubescens* was extracted using an Eastep^®^ Super total RNA Extraction Kit (Promega, Shanghai, China). First-strand cDNAs were synthesized using FastKing One-Step RT-PCR Kit (TIANGEN Biotech, Beijing, China) for amplification of putative flavonoid *E. pubescens* prenyltransferase genes (*EpPT*s). Specific primers (**Table S7**) were designed according to the candidate gene sequences and nested PCR amplification was performed using Q5^®^ High-Fidelity DNA Polymerases (New England BioLabs, MA, USA). The PCR program was set up as follows: denaturation at 98°C for 30 s; 35 cycles of 98°C for 10 s, 53-56°C for 30 s, 72°C for 1 min and 20 s; and a final extension at 72°C for 5 min. After the first-round PCR amplification, PCR products were used as the template for second-round PCR amplification under the same PCR conditions. PCR products were purified using AxyPrep DNA Gel Extraction Kit (Corning, NY, USA) and further cloned into pTOPO-Blunt simple vectors (LANY, Beijing, China), which were transformed into *Escherichia coli* competent cell *Trans*1-T1 (TransGen Biotech, Beijing, China) and confirmed by Sanger sequencing. Candidate *EpPT* genes and two truncated *EpPT*ΔTP constructs were cloned into the entry vector pENTR/D-TOPO (Invitrogen, CA, USA) and confirmed by Sanger sequencing, and then they were inserted into yeast expression vectors (pDR196GW) using LR Clonase™ II Enzyme (Invitrogen, CA, USA). The resulting vectors, pDR196GW-*EpPT* and pDR196GW-*EpPT*ΔTP were separately transformed into yeast strain DD104 using the modified LiAc method (Pompon et al., 1996; Liu et al., 2003). The transformants were screened on SD/-Ura plates and confirmed by PCR. Yeast expression vector pDR196GW and strain DD104 were kindly provided by Professor Guodong Wang (Institute of Genetics and Developmental Biology, the Chinese Academy of Sciences, Beijing, China). Yeast-Extract Peptone Adenine Dextrose Medium (YEPAD) and Ura Minus Medium were purchased from FunGenome (Beijing FunGenome Co. Ltd, Beijing, China).

Monoclonal yeast clones, which contained empty pDR196GW vector (blank control) or pDR196GW-*EpPTs*/*EpPT*ΔTPs vector, were cultured in 5 mL of SD (-Ura) overnight at 28°C, separately. Cultured yeast of 200 μL was inoculated into 780 μL of SD (-Ura) broth supplemented with 17 flavonoid substrates (**Figure S3**) with a final concentration of 200 μM and grown at 28°C for 72 h. These flavonoid substrates were purchased from Shanghai Yuanye Bio-Technology Co. Ltd (Shanghai, China). After the incubation period, the enzymatic reaction mix was ultrasonically extracted three times with an equal volume of ethyl acetate for 20 min. The ethyl acetate solvent in samples was evaporated and the dried powder was then dissolved in 200 μL absolute methanol for HPLC and UHPLC-PDA-Q-TOF/MS analyses.

### HPLC purification, LC-MS/MS and ^1^H NMR methods for reaction products

The yeast cultures fed with kaempferol were scaled up to 1.5 L, and extracted with ethyl acetate. For the isolation of enzymatic products, semipreparative RP-HPLC was conducted on a Lumtech K-501 equipped with a YMCPack ODS-A column (250 mm × 10 mm i.d., 5 mm, YMC Co., Ltd., Kyoto, Japan) at a flow rate of 3 mL·min^-1^. The solvent system consisted of a linear gradient (70%–100%, v/v) of methanol in water over 0-20 min. UV detection was set at 254 nm and 280 nm.

LC-MS/MS analysis was performed on Waters Xevo G2-XS Tof (Waters, Milford, MA, USA). The separation was carried out with a Waters ACQUITYTM HSS T3 C18 column (2.1 mm×100 mm, 1.8 μm) at 40°C. The gradient is consisted of 0.1% formic acid (A) and acetonitrile (B) as the mobile phase, 0-1.5 min (21%-24% B), 1.5-3 min (24%-25% B), 3-4 min (25%-29% B); 4-5 min (29% B); 5-6.5 min (29%-32% B); 6.5-7 min (32%-44% B); 7-8 min (44%-45% B); 8-9 min (45%-46% B); 9-11min (46%-95% B). The operating conditions were as follows: flow rate of 0.6 mL·min^-1^ with positive ion ESI mode, a capillary voltage at 3 kV, a cone voltage at 50 V, desolvation gas with a flow rate of 850 L·h^-1^. The mass-to-charge ratio was scanned from 100 to 1,600 *m/z*.

Approximately 10 mg of each compound was evaporated to dryness under N_2_ gas, resuspended in dimethyl sulfoxide-*d*_6_ (DMSO-*d*_6_), and analyzed through ^1^H NMR spectra acquired on a Bruker 600 spectrometer (Bruker, Rheinstetten, Germany).

^1^H NMR data of substrate and prepared isoprene products were shown as follows:

**Kaempferol (1).** ^1^H NMR (DMSO-*d*_6_, 600 MHz): *δ*_H_ 6.19 (1H, d, *J*=2.6 Hz, H-6), 6.44 (1H, d, *J*=2.4 Hz, H-8), 6.93 (2H, d, *J*=9.0 Hz, H-3′ and H-5′), 8.05 (2H, d, *J*=9.0 Hz, H-2′ and H-6′), 9.44 (1H, s, 3-OH), 10.10 (1H, s, 4′-OH), 10.80 (1H, s, 7-OH), 12.50 (1H, s, 5-OH).
**8-prenylkaempferol (1a)**. ^1^H NMR (DMSO-*d*_6_, 600 MHz): *δ*_H_ 1.63 (3H, s, H-5′′), 1.75 (3H, s, H-4′′), 3.43 (2H, d, *J*=7.0 Hz, H-1′′), 5.17 (1H, t, *J*=6.9 Hz, H-2′′), 6.29 (1H, s, H-6), 6.93 (2H, d, *J*=9.0 Hz, H-3′ and H-5′), 8.04 (2H, d, *J*=9.0 Hz, H-2′ and H-6′), 12.42 (1H, s, 5-OH).

## Results

### Chromosome-level genome assembly of *E. pubescens*

Both previous studies and our survey showed that the genome size of *Epimedium* species varied from 3.14 Gb/1C to 4.49 Gb/1C, and the size of *E. pubescens* genome was estimated to be 3.23 Gb based on k-mer (k=23) distribution analysis and flow cytometry analysis (Chen et al., 2012; Liu et al., 2013; Zhang et al., 2018) (**Table S1**). In addition, the k-mer analysis of *E. pubescens* further revealed a relatively low hybridization rate at 1.2% and an estimated percentage of repetitive elements at 61.57%, suggesting that *E. pubescens* possessed a relatively noncomplicated genome (the *E. pubescens* genome is complicated compared to model diploid plants such as *Arabidopsis thaliana* and *Oryza sativa*, but uncomplicated compared to allopolyploid plants *Triticum aestivum* and *Medicago sativa*) (Michael, 2014). Therefore, *E. pubescens* was eventually selected as a representative species for the construction of reference genome for *Epimedium* genus.

**Figure 2:**
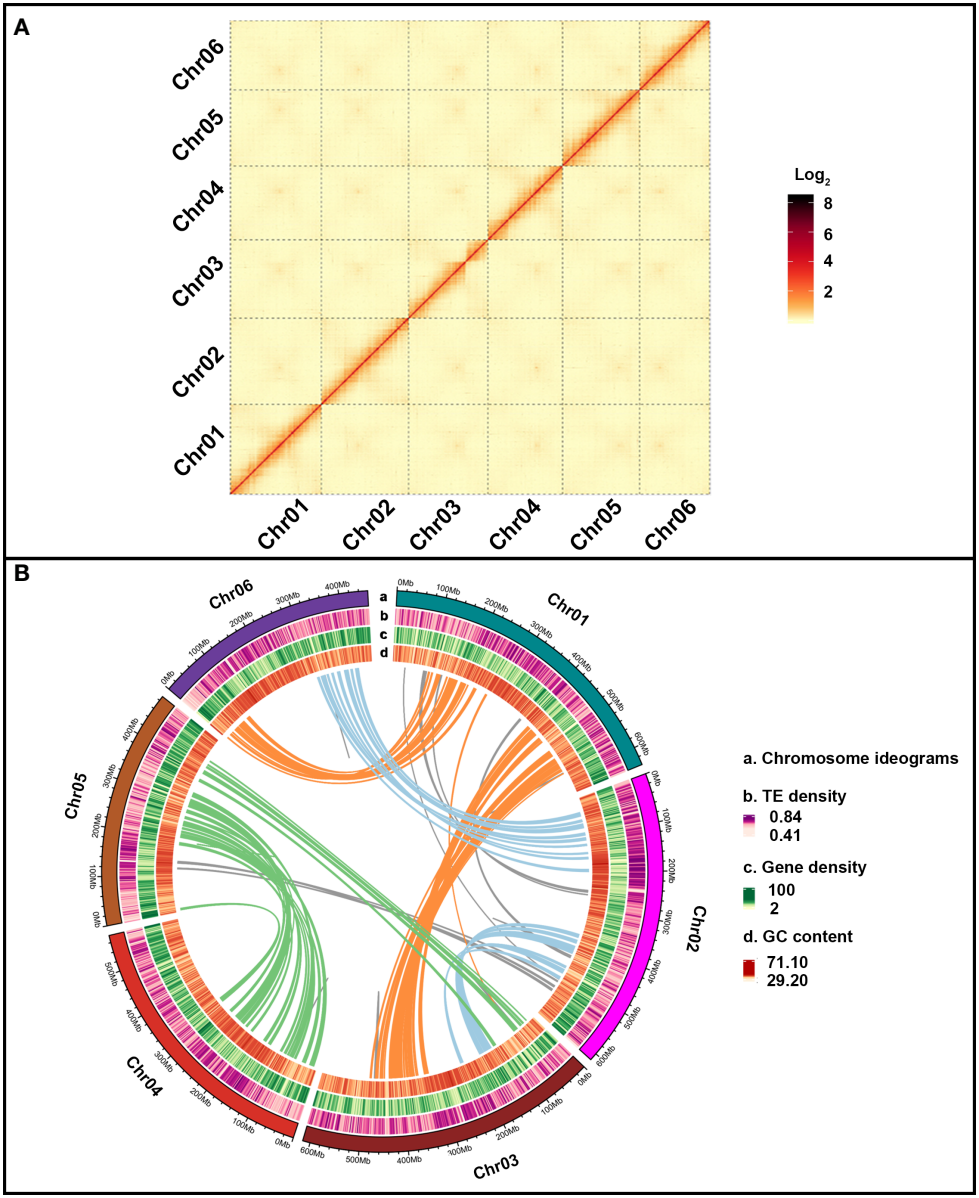
*E*. *pubescens* genome assembly. **(A)** Dotplot of *E*. *pubescens* syntenic blocks from Hi-C (High-throughput chromosome conformation capture) genome assembly. Each red segment was marked by different pseudo-chromosome label on the X and Y axis. **(B)** Circos graph of *E*. *pubescens* genome assembly. From outer to inner circles, the outer circle of (a) illustrated 6 chromosomes of *E*. *pubescens*; the inner circles of (b–d) showed TE density, gene density and GC content. The solid color lines inside all the circles represented segmental duplication relationships between syntenic blocks of different chromosomes, Chr01-Chr06 and Chr01-Chr03 with orange lines, Chr02-Chr06 and Chr02-Chr03 with light blue lines, Chr03-Chr05 and Chr04-Chr05 with green lines and others with grey lines.

Approximately 48.01 million Oxford Nanopore Technology (ONT) long reads were acquired and accounted for 906 Gb (271.32×) with an average read size of 20,267 bp (**Table S3**, **S4**). The preliminary genome assembly of *E. pubescens* was created with 39,251,621 ONT reads of >2 kb in length, yielding 6,229 contigs with a total size of 3.34 Gb (**Table S8**). Illumina reads of 771 Gb (230.94×) was used for further polishing the initial genome assembly to achieve contig N50 of 871.79 kb (**Table 1** and **Table S8**). Subsequently, 1,965,567,758 paired-end reads from the Hi-C (High-throughput chromosome conformation capture) sequencing were used to successfully anchor 6,176 contigs (99.15%) onto six chromosome-level pseudo-molecules, with an average size of 538.98 Mb (**Figure 2A** and **Table S9**). The resultant chromosome-level genome assembly had a size of 3.34 Gb with evenly distributed genes, slightly unevenly distributed Transposable Elements (TEs) and 37.48% of average GC content (**Table 1** and **Figure 2B**). Eventually, 5,162 contigs of 3.16 Gb were fully anchored and oriented on six complete chromosome-level pseudo-molecules (**Table 1** and **Figure 2B**).

**Table 1:** Assembly and annotation statistics of *E. pubescens* genome.

Genome assembly feature	Number	Size/Percentage
Estimated genome size		3.23 Gb
Assembled sequences		3.34 Gb
Total contigs	6229	3.34 Gb
N50 contigs		871.79 kb
Anchored and oriented contigs	5162	3.16 Gb
Average pseudo-chromosome size		538.98 Mb
Gaps	5156	0.49 Mb
GC content		37.48%
Repeat percentage		66.93%
Protein-coding genes	44,722	487.81 Mb
Average length of protein-coding genes		4.53 kb

Homology and *ab initio* based gene prediction strategies were combined to predict 44,722 protein-coding gene models from *E. pubescens* genome assembly (**Table 1**). Among these gene models, protein coding genes accounted for 487.81 Mb (14.61%) with an average gene length of 13.36 kb (**Table 1**), and have 33,355 with 1~5 exons (74.58%) and 11,367 with more than 5 exons (25.40%) (**Table S10**). A total of 94.5% gene models were successfully annotated by eight protein databases (**Table S5**), and a total of 8,415 noncoding RNA genes were identified, including 1,713 rRNA (ribosomal RNA), 172 miRNA (microRNAs), 5,794 tRNA (transfer RNA), 278 snoRNA (small nucleolar RNA), and 458 snRNA (small nuclear RNA) (**Table S11**). In addition, large amounts of repetitive elements were identified in the *E. pubescens* genome (66.93%), which was relatively high in comparison with other species of Ranunculales (**Table S12**). Among these repetitive elements, there were 62.61% Class I retrotransposons, which were predominantly the long terminal repeats (LTRs) with mainly Gypsy and Copia at 26.62% and 5.03% respectively, and 7.92% of DNA transposons (Class II) (**Table S12**, **S13**). Notably, 1,236 out of 1,375 (89.9%) BUSCO (Benchmarking universal single-copy orthologues) core genes were confirmed in the final chromosome-level *E. pubescens* genome (**Table S14**). These above evidences indicated that the current chromosome-level genome assembly of *E. pubescens* was relatively complete and accurate.

### Comparative genomic analyses revealed the lacking of WGT-γ

**Figure 3:**
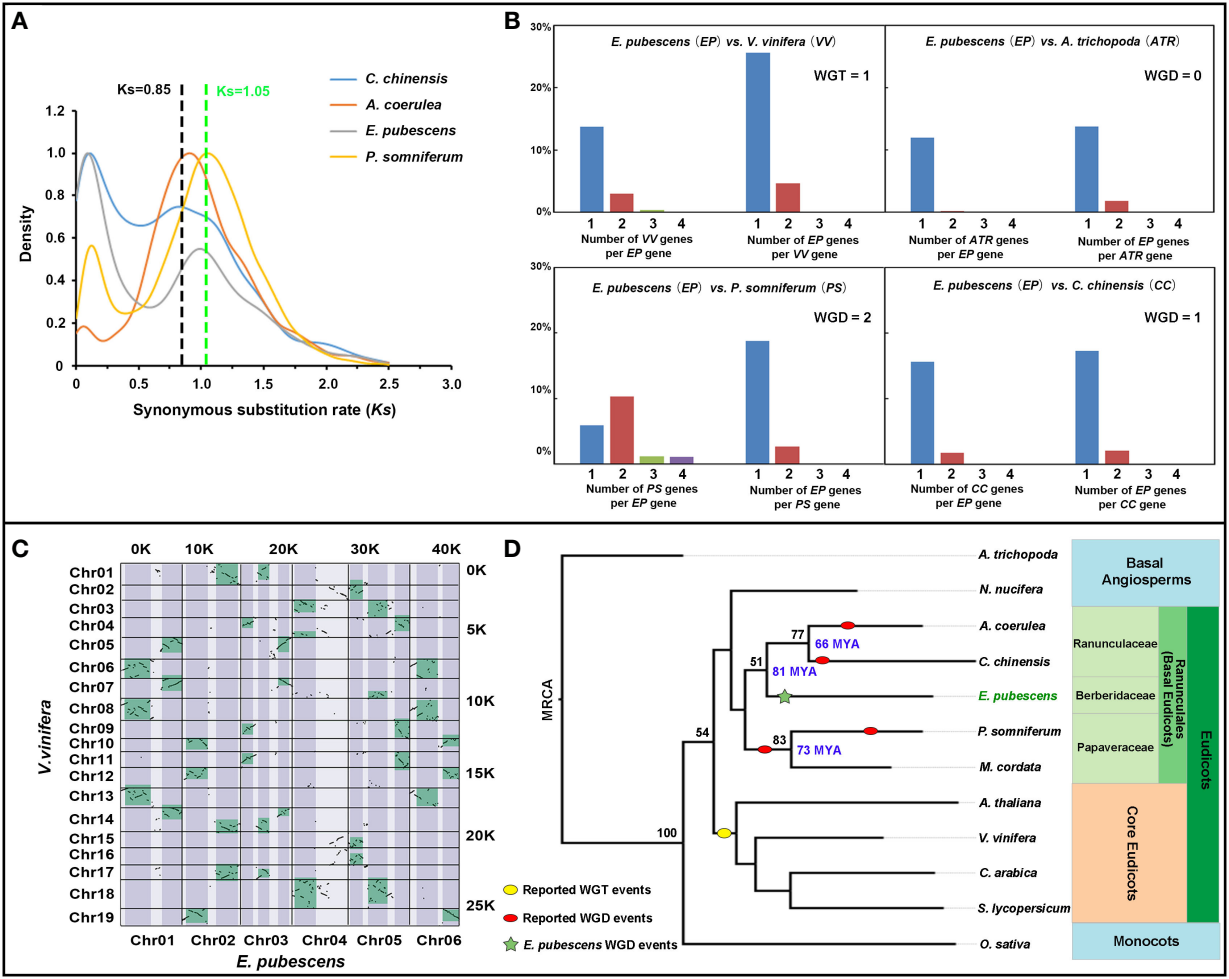
Comparative genomic analysis of *E. pubescens* and other species. **(A)** Ks plots of orthologous genes in the genome of *C*. *chinensis*, *A. coerulea*, *E*. *pubescens*, and *P. somniferum*. Blue, red, light purple and yellow curves represent *C*. *chinensis*, *A. coerulea*, *E*. *pubescens* and *P. somniferum* respectively. **(B)** Comparison of orthologous gene ratio of *E*. *pubescens* to *Vitis vinifera, P. somniferum*, *Amborella trichopoda*, and *C*. *chinensis*, reciprocally. Blue, red, green and purple bars represent one, two, three and four orthologous genes. Short names were assigned as *EP* (*E. pubescens*), *VV* (*V. vinifera*), *ATR* (*A. trichopoda*), *PP* (*P. somniferum*) and *CC* (*C. chinensis*). Top left is the comparison of orthologous gene ratio of *EP* to *VV*; top right, the comparison of orthologous gene ratio of *EP* to *ATR*; bottom left, the comparison of orthologous gene ratio of *EP* to *PP*; bottom right, the comparison of orthologous gene ratio of *EP* to *CC*. **(C)** Dotplot of syntenic blocks between *E*. *pubescens* and *V. vinifera*. Chromosomes of *V. vinifera* and *E*. *pubescens* were labeled on the Y-axis and X-axis respectively; the number of syntenic blocks from *V. vinifera* and *E*. *pubescens* was labeled on the right and top respectively; syntenic blocks with 3:1 ratio of *V. vinifera* to *E*. *pubescens* were green-colored on the dotplot. **(D)** Phylogenetic tree of 12 plant species. In phylogenetic tree, red and yellow ovals represent the reported WGD events and WGT-γ event, respectively; the green star highlighted the independent WGD event in *E*. *pubescens*; ML (Maximum Likelihood Method) bootstrap values were labeled on the left of key phylogenetic tree nodes in black and the divergence times were labeled on the right of key phylogenetic tree nodes in blue, based on Timetree online services. Based on APG IV system, the phylogenetic classification of 12 plant species was labeled on the right of phylogenetic tree with different background color.

The genomic syntenic blocks and orthologous gene ratio between *V. vinifera* and *E. pubescens* was analyzed, demonstrating that there was a significant percentage of genes with 3:1 ratio of *V. vinifera* to *E. pubescens*, but not be observed between *P. somniferum*/*C. chinensis* and *E. pubescens* (**Figures 3B, C** and **Figure S1A–C**). It was known that only one Whole Genome Triplication (WGT-γ) event occurred in *Vitis vinifera* (Jaillon et al., 2007), and moreover, *P. somniferum* and *C. chinensis* were also proven to have not experience WGT-γ event (Guo et al., 2018; Liu et al., 2021c). The combination of previous research results with current evidences suggested that *E. pubescens* had escaped the ancient WGT-γ event. To further dissect genome evolutionary history, the analysis of synonymous substitution rate per synonymous site (Ks) was performed among the orthologous genes of *E. pubescens*, *P. somniferum*, *Coptis chinensis* and *Aquilegia coerulea* from Ranunculales, revealing an significant peak (around Ks=0.997) in *E. pubescens* compared to *C. chinensis* (around Ks=0.85) (**Figure 3A**), indicating that only one Whole Genome Duplication (WGD) event occurred during *E. pubescens* genome evolution, which was consistent with the widespread occurrence of WGD events in flowering plants (Jaillon et al., 2007).

To investigate the evolving history of *E. pubescens*, a phylogenetic tree was constructed using shared single-copy orthologues identified by Orthofinder from *E. pubescens* and 11 key angiosperm species, including *A. trichopoda*, *P. somniferum*, *C. chinensis* and *V. vinifera*, etc. (**Figure 3D** and **Table S6**). In the resultant phylogenetic tree, *E. pubescens* and other species from Ranunculales formed an early diverging taxonomic clade of basal eudicots, and these species were further divided into 3 distinct taxonomic groups, including Ranunculaceae (*A. coerulea* and *C. chinensis*), Papaveraceae (*P. somniferum* and *Macleaya cordata*) and Berberidaceae (*E. pubescens*). Using Treetime online services, the divergence time between Ranunculaceae and Berberidaceae was determined at ~81 MYA (million years ago) and the formation of Ranunculaceae was also estimated at ~66 MYA. In addition, there exhibited some strong patterns of inter-chromosomal synteny between chromosomes of *E. pubescens*, as shown in **Figure S1C**, inferring that multiple ancient chromosomal breaks and fusion events could occur after the only WGD event in *E. pubescens* genome.

### Chromosome-level genome guided the identification of a prenyltransferase gene

**Figure 4:**
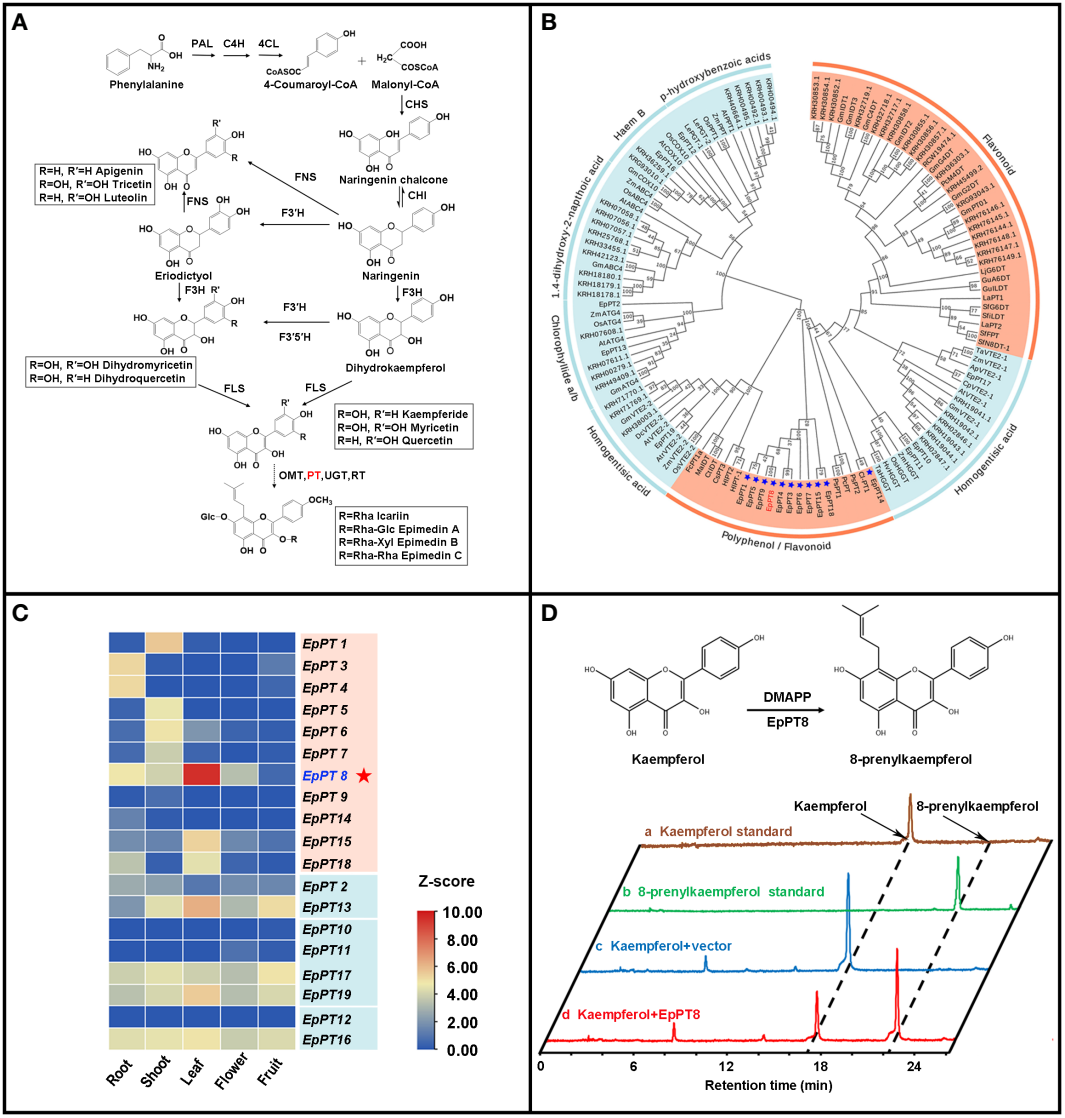
Analysis of specific prenyltransferase genes for biosynthesis of prenylated flavonoids. **(A)** Biosynthetic pathways of prenylated flavonoids in *E*. *pubescens*. **(B)** Phylogenetic tree of prenyltransferase genes (PTs). Clades of PTs were classified by their respective substrates, including two categories, flavonoid/polyphenol and others labeled with coral color and light blue color respectively; 11 candidate flavonoid PTs from E pubescens were marked with blue star and EpPT8 was highlighted with red color. **(C)** Expression profiles of 19 putative *E*. *pubescens* prenyltransferase genes (EpPTs) in different tissues. Expression levels in the heatmap were scaled from blue to red (low to high); protein IDs were labeled on the right of heatmap; different tissue samples were labeled at the bottom. **(D)** Verification of the products from the reaction catalyzed by recombinant EpPT8 protein using kaempferol as substrate. The reaction catalyzed by EpPT8 was illustrated on the top part of the panel; at the bottom part of the panel, HPLC chromatographs at 254 nm from the examined standards and different reaction mixes were colored with gold, green, blue and red color, representing Kaempferol standard, 8-prenylkaempferol standard, the reaction mix of Kaempferol+vector and the reaction mix of Kaempferol+EpPT8 respectively; the peaks of Kaempferol and 8-prenylkaempferol were pointed out on the first chromatograph with black arrows separately and the corresponding peaks of other three chromatographs were indicated through black dotted line.

It has been known that, for *Epimedium* flavonoids, after the formation of basic flavonoid skeleton, several enzymatic steps still are needed for further modification, including prenylation, methylation and glycosylation (Pandey et al., 2016; Nabavi et al., 2020). The addition of prenyl group to flavonoids is one of the most important enzymatic steps, which is catalyzed by prenyltransferases (PTs) (**Figure 4A**), and provides the medicinal efficacy for *Epimedium* flavonoids (Ming et al., 2013; Mbachu et al., 2020). Genome-wide homology-based search discovered 19 potential *PT*s from UbiA (ubiquinone biosynthesis gene A) superfamily in *E. pubescens* (**Figure 4B** and **Table 2**). Subsequently, a phylogenetic tree was constructed with these 19 EpPTs and additional 108 UbiA PTs from other plant species (**Table 2** and **Table S15**), revealing that all PTs of UbiA superfamily could be clustered into distinctive groups based on the preference of their substrates, such as flavonoids, polyphenol, chlorophyllide a/b and homogentisate acid etc. (**Figure 4B**). It was found that, 11 EpPTs formed a distinctive cluster that was nested in the clade of PTs using polyphenols or flavonoids as preferred substrates, and six out of these 11 *EpPT*s (*EpPT3*, *EpPT4*, *EpPT5*, *EpPT6*, *EpPT7*, *EpPT8* and *EpPT9*) were found to form a cluster at the end of Chr02 (data not shown).

**Table 2:** Characteristics of 19 *EpPT*s.

Gene name	Substrates	Chromosome	Predicted protein size
*EpPT1*	Flavonoid	Chr01	392
*EpPT3*	Flavonoid	Chr02	265
*EpPT4*	Flavonoid	Chr02	394
*EpPT5*	Flavonoid	Chr02	155
*EpPT6*	Flavonoid	Chr02	229
*EpPT7*	Flavonoid	Chr02	261
*EpPT8*	Flavonoid	Chr02	391
*EpPT9*	Flavonoid	Chr02	379
*EpPT14*	Flavonoid	Chr03	266
*EpPT15*	Flavonoid	Chr03	397
*EpPT18*	Flavonoid	Chr06	398
*EpPT2*	Chlorophyllide a/b	Chr01	374
*EpPT13*	Chlorophyllide a/b	Chr03	424
*EpPT10*	Homogentisate acid	Chr02	229
*EpPT11*	Homogentisate acid	Chr02	415
*EpPT17*	Homogentisate acid	Chr05	407
*EpPT19*	Homogentisate acid	Chr06	399
*EpPT12*	Haem B	Chr03	222
*EpPT16*	Haem B	Chr04	447

EpPTs, *E. pubescens* prenyltransferases.

To further explore these 11 *E. pubescens PT*s, their tissue expression profiles were analyzed, showing that *EpPT8* was most highly expressed in leaves, where the bioactive compounds accumulated (**Figure 4C**) (Guo and Xiao, 1996; Zhou et al., 2012). Further analysis revealed that *EpPT8* shared relatively high sequence similarity with known plant flavonoid *PT* genes (**Figure S2**), suggesting that *EpPT8* was a promising candidate gene for the addition of prenyl group on flavonoids in *E. pubescens*. In *EpPT8*, the transit peptides were predicted to be 32 or 82 amino acids in length at the N terminus which were subsequently removed to generate the truncated *EpPT8* constructs. Yeast strain DD104 cells were transformed with *EpPT8* and two truncated *EpPT8* constructs, and subsequently co-cultured with 17 representative flavonoid substrates listed in **Figure S3**. After purification, the reaction mixtures were subjected to the examination of PT enzymatic activities. Notably, the recombinant EpPT8 proteins only showed enzymatic activity towards kaempferol (highest enzymatic activity), apigenin and quercetin out of 17 substrates (**Figure S3**). Meanwhile, the other two truncated EpPT8 proteins have similar levels of prenylation activities (**Table 3**), suggesting that putative transit peptides in EpPT8 did not affect the enzymatic activity of EpPT8 proteins.

**Table 3:** Results of enzymatic reactions.

Substrate category	Substrate names	EpPT8	EpPT8△TP32	EpPT8△TP82
Flavonol	Kaempferol	+	+	+
	Quercetin	+	+	+
	Kaempferide	ND	ND	ND
	Tamarixetin	ND	ND	ND
	Myricetin	ND	ND	ND
	Mearnsetin	ND	ND	ND
Flavone	Apigenin	+	+	+
	Luteolin	ND	ND	ND
	Tricin	ND	ND	ND
	Tricetin	ND	ND	ND
Dihydroflavonol	Dihydrokaempferol	ND	ND	ND
	Dihydroquercetin	ND	ND	ND
	Hydromyricetin	ND	ND	ND
Dihydroflavone	Liquiritigenin	ND	ND	ND
	Naringenin	ND	ND	ND
	Eriodictyol	ND	ND	ND
Chalcone	Naringenin chalcone	ND	ND	ND

ND means "Not detectable".

**Figure 5:**
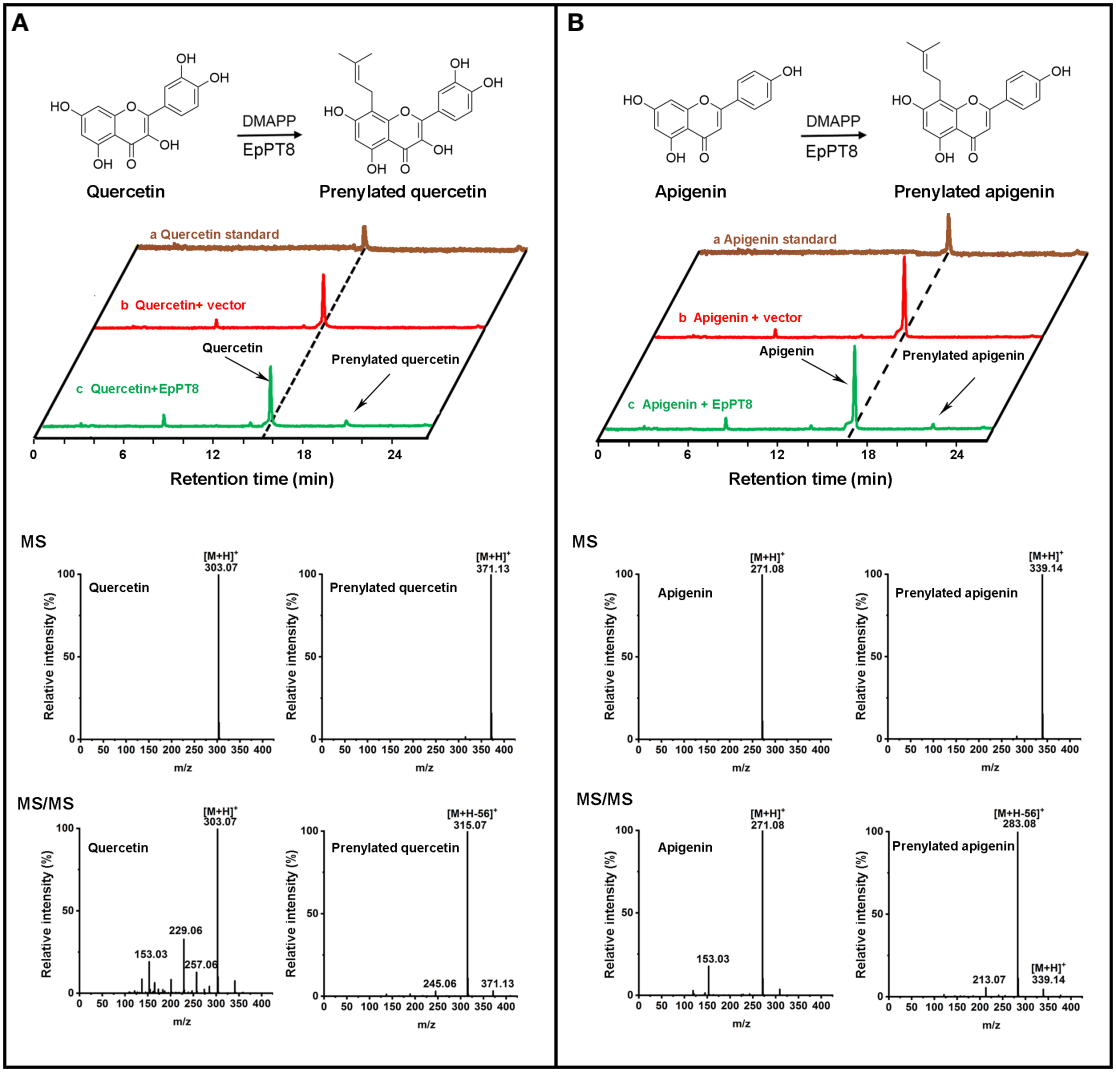
Determination of the enzymatic products of the recombinant EpPT8 protein with quercetin and apigenin as substrates. **(A)** The reaction with quercetin catalyzed by the recombinant EpPT8 protein. The HPLC chromatograph of reaction mix was shown in the upper panel; in the lower panel, MS (upper) and MS/MS (lower) chromatograms at 270 nm of quercetin and prenylated quercetin was on the left and right, respectively. **(B)** The reaction with apigenin catalyzed by the recombinant EpPT8 protein. The HPLC chromatograph of reaction mix was shown in the upper panel; in the lower panel, MS (upper) and MS/MS (lower) chromatograms of apigenin and prenylated apigenin were on the left and right, respectively.

To examine the products in the EpPT8 catalyzed reaction using kaempferol as substrate, their HPLC chromatographs, MS spectra and MS/MS spectra were acquired for the confirmation of chemical property. The above reaction products were shown at about 55% conversion ratio of substrate to product and possessed the same HPLC retention time and reference data as 8-prenylkaempferol standard (**Figure 4D**, **Figure S3**, **S4**) (Liu et al., 2021b), indicating that EpPT8 was able to prenylate kaempferol at C-8 position. To confirm the chemical structure, the EpPT8 catalyzed reaction products were further separated with preparative liquid chromatograph and determined by NMR experiments. In ^1^H NMR spectrum (**Figure S5**), the characteristic signals (Hillerns and Wink, 2005; Kim et al., 2018) were identified as follows: *δ*_H_=1.63 ppm (3H, s, H-5′′) and *δ*_H_=1.75 ppm (3H, s, H-4′′) for two methyl groups, *δ*_H_=3.43 ppm (2H, d, *J*=7.0 Hz, H-1′′) for one methylene and *δ*_H_=5.18 (1H, t, *J*=6.9 Hz, H-2′′) for one methine, suggesting the addition of a dimethylallyl moiety onto kaempferol (De Souza et al., 2017). By comparing the ^1^H NMR spectrum of kaempferol (De Souza et al., 2017) with that of 8-prenylkaempferol in the literature (Hillerns and Wink, 2005), the addition of dimethylallyl unit was determined to occur at the C-8 position (**Figure S5**). Taken together, EpPT8 could catalyze the prenylation of kaempferol, leading to the principal enzymatic product, 8-prenylkaempferol (**Figure 4D**). Moreover, the enzymatic products of EpPT8 with quercetin and apigenin as substrates were also predicted to be prenylated products with expected MS and MS/MS spectrum (**Figures 5A, B**), but the prenylation position could not be resolved by NMR due to an extremely low amount of available enzymatic products. In addition, among 11 PTs, EpPT1, EpPT4 and EpPT9 were close to full length with minor change (similarity 88.52%-95.4%) compared to EpPT8, but they are either failed to be cloned or proved no PT activity. The rest of the PTs are with low similarity to EpPT8 and either N-terminal or C-terminal truncated.

## Discussion

Consistent with the previous reports, the current genome assembly of *E. pubescens* possessed a large genome size of 3.34 Gb (the most frequently observed published genome size is around 500 Mb) (Michael, 2014). Our chromosome-level genome assembly of *E. pubescens* with contig N50 of 871.79 kb was substantially better than those of other Ranunculales plant genomes (Rounsaville and Ranney, 2010; Liu et al., 2017b; Guo et al., 2018). Moreover, the quality of current genome assembly was further manifested by the relatively high ratio of mapped BUSCO core genes and successfully applied to whole genome search for prenyltransferase (PT) genes. Based on the comparative analyses of *E. pubescens* genome, as other species of Ranunculales already exhibited (Liu et al., 2017b; Filiault et al., 2018; Guo et al., 2018; Liu et al., 2021c), *E. pubescens* escaped the early WGT-γ event, which is the key to the expansion of core eudicots. Specially, the genome evolution of *E. pubescens* was also involved in an early WGD event between 66 and 81 MYA.

Notably, there is a large discrepancy between the limited number of chromosomes and the large genome size of *Epimedium* species (Chen et al., 2012; Liu et al., 2013; Wang et al., 2020). The *E. pubescens* genome is consisted of 66.94% of repetitive elements, which is much higher than other species from Ranunculales. Among these repetitive elements, a large portion of LTR (60.08%) was identified and may be considered as one of the major reasons for a large discrepancy between few of chromosomes and such large genome size in *E. pubescens* (Chen et al., 2012; Novikov et al., 2012) For example, maize B73 genome with a size of 2.1 Gb contains a total of 64% repetitive elements with 59.98% of LTR, which is proven to largely contribute to its large genome size (Jiao et al., 2017). Based on the Large Genome Constraint Hypothesis (LGCH), the species with large genomes have some constraints on plant performance such as reducing maximum photosynthetic rates (Meyerson et al., 2020). In general, it is imperative for *E*. *pubescens* to rapidly adapt to a variety of natural environmental changes by maintaining enormous molecular and phenotypic diversification from a great amount of LTR and the large size of gene introns (Chen et al., 2012; Novikov et al., 2012). The above genomic characteristics might partly explain why *Epimedium* plants are not resistant to sunlight and high temperature, and have to grow in the shade area (Ma et al., 2011; Liu et al., 2017a).

In recent studies, there are more and more evidences confirming the 8-prenylated flavonoids as the key medicinal components of Herba Epimedii (Wang et al., 2019; Guo et al., 2020). PTs are a class of enzymes responsible for prenyl moiety transferring, which is the key biosynthetic step of prenylated flavonoids, such as 8-prenylkaempferol in *E. pubescens* (Yang et al., 2015; Wang et al., 2021). In *E. pubescens* genome, we were able to identify 19 putative *E. pubescens PT* genes (*EpPT*s) on six chromosomes and two contigs (**Table 2**). The phylogenetic analysis demonstrated that 11 flavonoid *EpPT*s formed a special cluster, which appeared to be unique to *E. pubescens*. Based on the patterns of tissue specific expression, *EpPT8* was identified as the possible PT to be further explored. LC-MS/MS and NMR experiments convincingly showed that EpPT8 possessed the activity for prenylation at C-8 position of kaempferol (**Figure 4D**). The successful cloning and characterization of *EpPT8* proved that the gene discovery strategy with the combination of genome, transcriptome and biochemistry approaches is feasible for the novel gene cloning and its functional characterization in *E. pubescens*.

Many Leguminosae PTs use isoflavonoids as the preferred substrates (Sukumaran et al., 2018), but EpPT8 from *E. pubescens* prefers kaempferol (flavonol) as the major substrate, which is consistent with the predominant accumulation of 8-prenylkaempferol derivatives in *E. pubescens* plants (Chen et al., 2015). The truncated EpPT8ΔTP32/82 proteins were found to possess similar levels of PT enzymatic activity (**Table 3**, **Figure S3**), suggesting that EpPT8ΔTP32/82 might be a more suitable choice for synthetic biology application than full length EpPT8. In addition, *EpPT8* was highly expressed in leaf tissue (**Figure 4C**), in which large amounts of 8-prenylkaempferol derivatives accumulates (Guo and Xiao, 1996; Zhou et al., 2012), implying a critical role of *EpPT8* for prenylated flavonols accumulation in *E. pubescens*.

Recent study showed that the recombinant LaPT2 protein from *Lupinus albus* (Leguminosae) could also use kaempferol as substrate to produce 8-prenylkaempferol, and possessed activity towards a wide range of flavonoid substrates, including flavonols, flavones, and naringenin, but *L. albus* plants only accumulate a trace amount of 8-prenylkaempferol in roots (Liu et al., 2021b). In a more recent study, the recombinant EsPT2 protein from *E. sagittatum* was found to be able to prenylate both kaempferol and methylated kaempferol (Wang et al., 2021). By contrast, our EpPT8 appeared to prefer kaempferol as the principal substrate (**Figure 4D**, **Figures 5A, B**), but only displaying negligible difference of enzymatic activity toward different flavonoid substrates comparing to EsPT2 (**Table S16**). The open reading frame of EpPT8 (1176 bp), EsPT2 (1176 bp), and LaPT2 (1209 bp) were predicted to encode 391, 391, and 402 amino acids, respectively. The EpPT8 shared 28% of amino acid sequence with LaPT2, and exhibited only 9 different amino acid residues from EsPT2. In summary, the prenylation specificity of homologues of EpPT8 might be differently evolved in diverse plant taxonomic groups.

In conclusion, a high-quality reference genome (3.34 Gb) of *E. pubescens* with a reliable annotation was constructed. Comparative genomic analysis revealed the absence of WGT-γ event shared in core eudicots, and further demonstrated the occurrence of one ancient WGD event between 66 and 81 MYA during the evolutionary history of *E. pubescens* genome. The cloning and characterization of *EpPT8* open a new door for improving the biosynthetic technique in the production of prenylated flavonoids, such as the precursors of icaritin and its derivatives.

## Data availability statement

The datasets presented in this study can be found in online repositories. The names of the repository/repositories and accession number(s) can be found in the article/**Supplementary Material**. All raw data were available at National Center for Biotechnology Information (NCBI) under project PRJNA747870; the genome assembly and annotation files are available at the National Genomics Data Center (NGDC, https://ngdc.cncb.ac.cn/gwh/Assembly/21845/show) under BioProject PRJCA006303.

## Author contributions

BG, GS, HZ, and YP contributed to conception and design of the study. CZ, GS, GM, XL, and YL organized the database. CX, GS, GD, GM, HZ, YL, and YW performed the statistical analysis. GS, HZ, YP, YL, YY, and YZ wrote the original draft of the manuscript. All authors contributed to manuscript revision, read, and approved the submitted version.

## Funding

This research was funded by the CAMS Innovation Fund for Medical Sciences (CIFMS) under Grant 2021-I2M-1-031 and 2017-I2M-3-013; the National Natural Science Foundation of China (31570306, U20A2004, 81473302); the Chongqing Science and Technology Commission under Grants cstc2018jcyjAX0316, cc-cstc-CA-19-2, cstc2019jcyj-msxmx0464, cstc2019jxjl-jbky10007, and cstc2020jxjl10004.

## Acknowledgments

We thank Professor Guodong Wang at Institute of Genetics and Developmental Biology, Chinese Academy of Sciences and Senior Engineer, Zhen Xue and Assistant Engineer Wangyin Yu at Institute of Botany, Chinese Academy of Sciences for the great help in the cloning of potential *Epimedium* PTs, the interpretation of LC-MS/MS and NMR data and data graphical presentation respectively in this project.

## Conflict of interest

The authors declare that the research was conducted in the absence of any commercial or financial relationships that could be construed as a potential conflict of interest.

## Publisher’s note

All claims expressed in this article are solely those of the authors and do not necessarily represent those of their affiliated organizations, or those of the publisher, the editors and the reviewers. Any product that may be evaluated in this article, or claim that may be made by its manufacturer, is not guaranteed or endorsed by the publisher.
