# Supplementary material for: The discovery of a key prenyltransferase gene assisted by a chromosome-level Epimedium pubescens genome
Source: Front Plant Sci. 2022 Nov 14;13:1034943. doi: 10.3389/fpls.2022.1034943 (PMC9702526; doi:10.3389/fpls.2022.1034943)
Supplement: Supplementary file 1 [file DataSheet_1.pdf]

Supplementary Material

Contents

1Supplementary Figures 1

Figure S1. Microsynteny analysis of *E. pubescens* and other plant genomes. 1

Figure S2. Multiple sequence alignment of the amino acid sequences from EpPT8 and representative plants PTs. 2

Figure S3. The chemical structures of flavonoid substrates used in the enzymatic assays. 3

Figure S4. Determination of enzymatic products of the recombinant EpPT8 protein by liquid chromatography mass and tandem mass spectrometry...4

Figure S5. 1H NMR spectrum of the enzymatic product of EpPT8 catalyzed reaction with kaempferol as substrate. 5

2 Supplementary Tables 6

Table S1. Karyotype and genome features of five *Epimedium* species...6

Table S2. The location record for wild populations of *E. pubescens* in China. 7

Table S3. Sequencing data used for *E. pubescens* assembly..8

Table S4. Summary of raw ONT data. 8

Table S5. Annotations of *E. pubescens* genome. 9

Table S6. Twelve plant species used in comparative genomic analyses. 10

Table S7. PCR primers for the amplification of different EpPT8 constructs. 10

Table S8. Summary of assembled contigs from ONT data. 11

Table S9. Characteristics of pseudo-chromosomes by Hi-C. 11

Table S10. Exon statistics from predicted gene models. 12

Table S11. Non-coding RNAs in *E. pubescens* genome. 12

Table S12. Identification of repetitive elements in *E. pubescens* genome. 13

Table S13. LTR copia and LTR gypsy in 6 pseudo-chromosomes. 15

Table S14. BUSCO evaluation of *E. pubescens* genome. 15

Table S15. PTs of the UbiA superfamily used in the phylogenetic tree. 16

Table S16. Comparison of enzymatic activity between EsPT2 and EpPT8................................18

1 Supplementary Figures

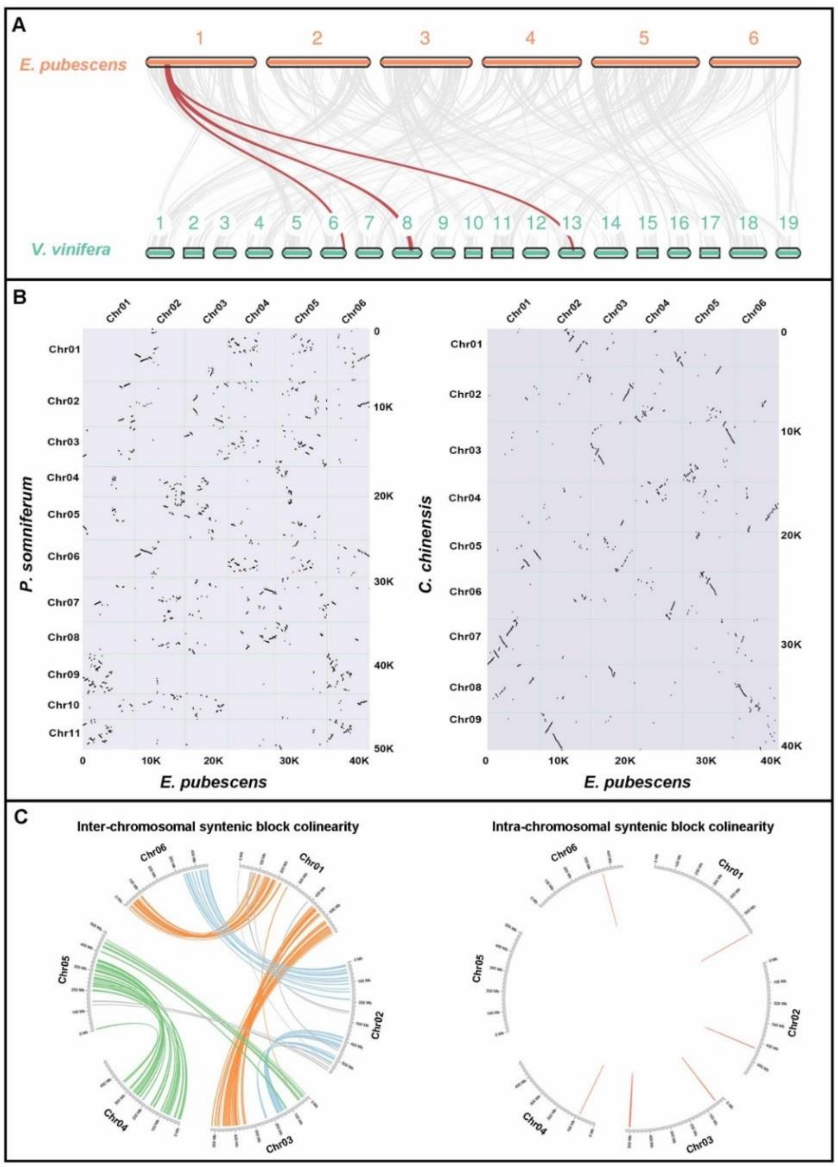

Figure S1. Microsynteny analysis of *E. pubescens* and other plant genomes. (A) Microsynteny analysis between *E. pubescens* and V. vinifera genome. The syntenic block on chromosome 1 of *E. pubescens* corresponds to three syntenic blocks on chromosomes 6, 8 and 13 of *V. vinifera*, respectively. (B) Dot plots of orthologues between *E. pubescens* and *P. somniferum* (left), and between *E. pubescens* and *C. Chinensis* (right). (C) Syntenic block collinearity plots. Left, inter-chromosomal syntenic block collinearity plot, Chr01-Chr06 and Chr01-Chr03 with orange lines, Chr02-Chr06 and Chr02-Chr03 with light blue lines, Chr03-Chr05 and Chr04-Chr05 with green lines; right, intra-chromosomal syntenic block collinearity plot, dark red lines for syntenic blocks on the same chromosome.

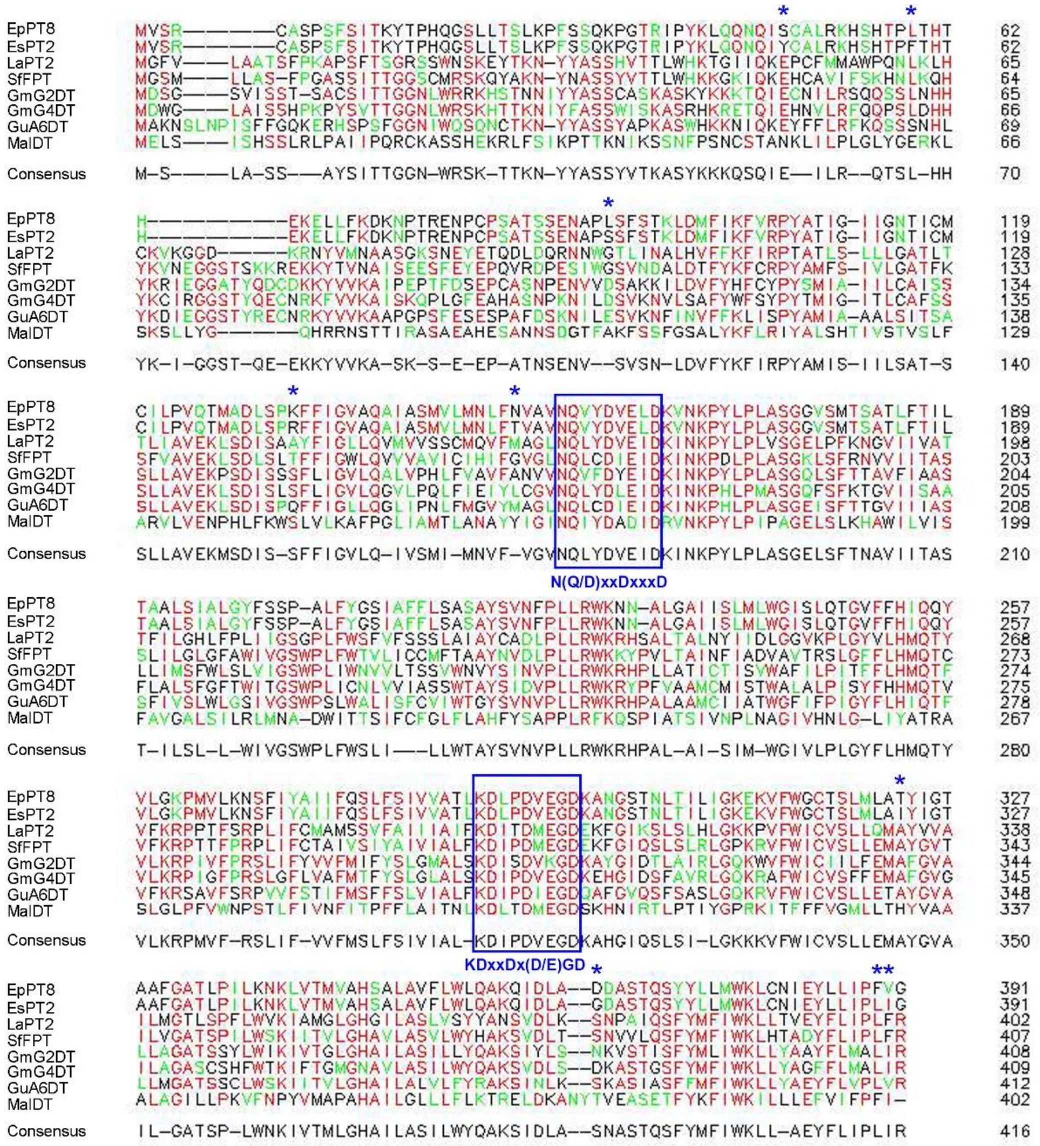

Figure S2. Multiple sequence alignment of the amino acid sequences from EpPT8 and representative plants PTs. The sequence similarity was illustrated by the sequence alignment and the amino acids were labeled with the colors of red, green and black (high, medium and low identity, respectively); the conserved motifs of PTs, N(Q/D)xxDxxxD and KDxxDx(D/E)GD were highlighted in blue boxes; the nine blue stars denote the difference of amino acid between EpPT8 and EsPT2; the representative plant PTs were described in Supplementary Table 15.

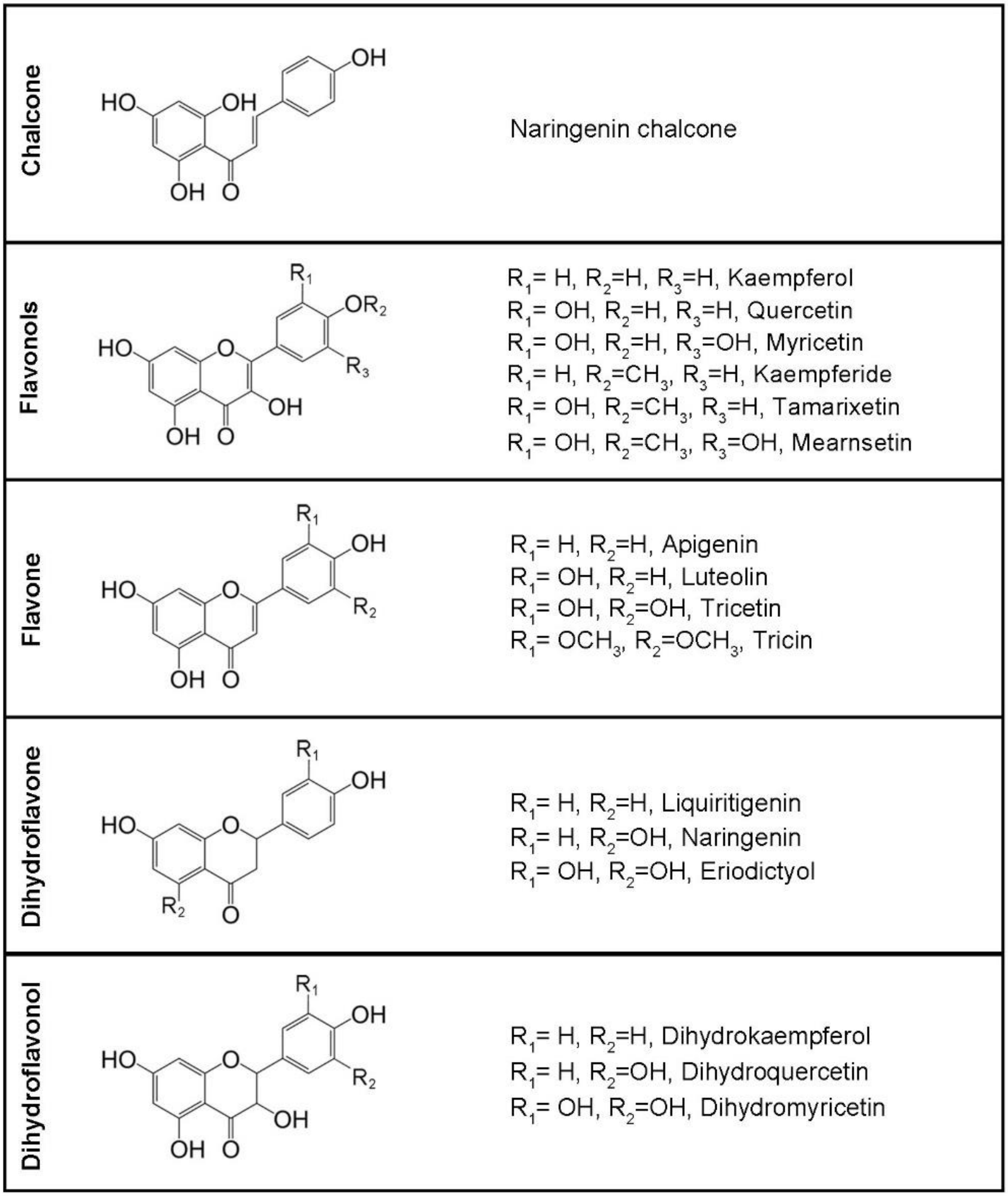

Figure S3. The chemical structures of flavonoid substrates used in the enzymatic assays. The classes and chemical structures of flavonoid substrates used in the enzymatic assays with the recombinant EpPT8 proteins expressed in yeast cells.

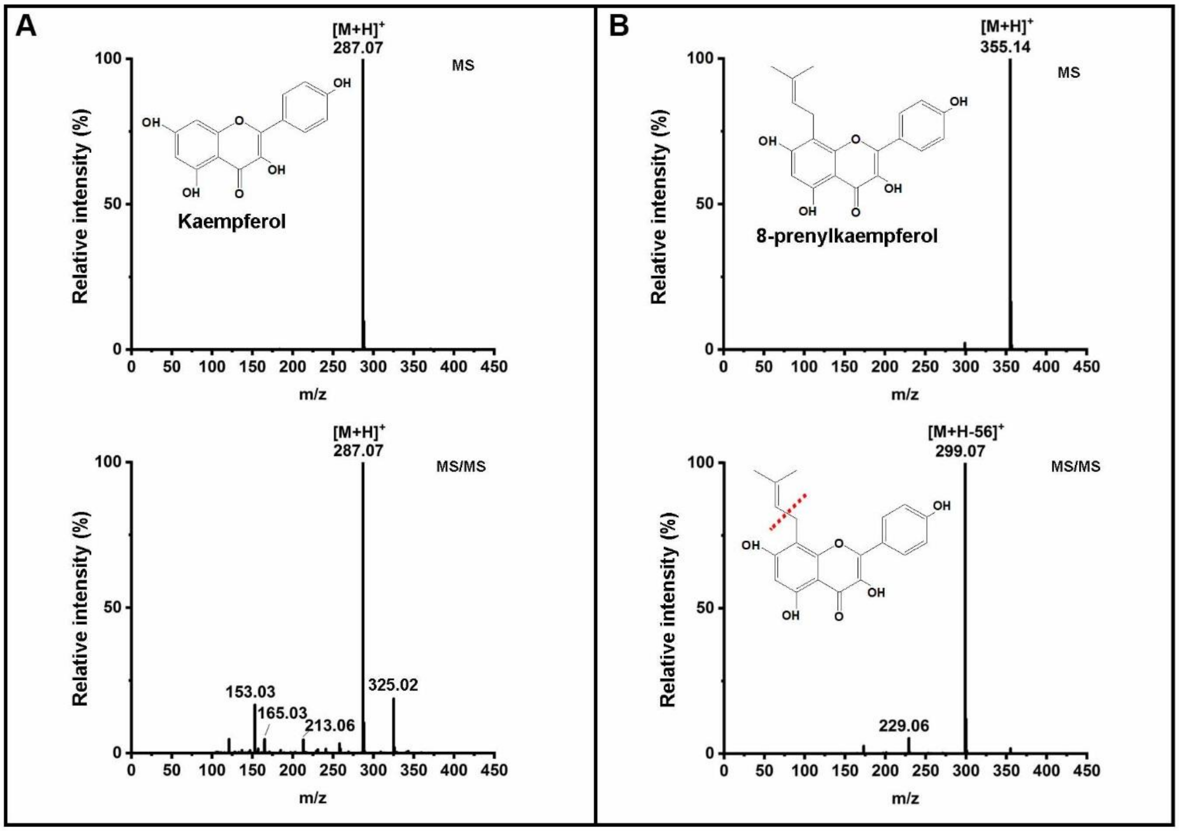

Figure S4. Determination of enzymatic products of the recombinant EpPT8 protein by liquid chromatography mass and tandem mass spectrometry. (A) The MS (upper) and MS/MS (lower) chromatograms of the substrate, kaempferol. (B) The MS (upper) and MS/MS (lower) chromatograms of the enzymatic product, 8-prenylkaempferol.

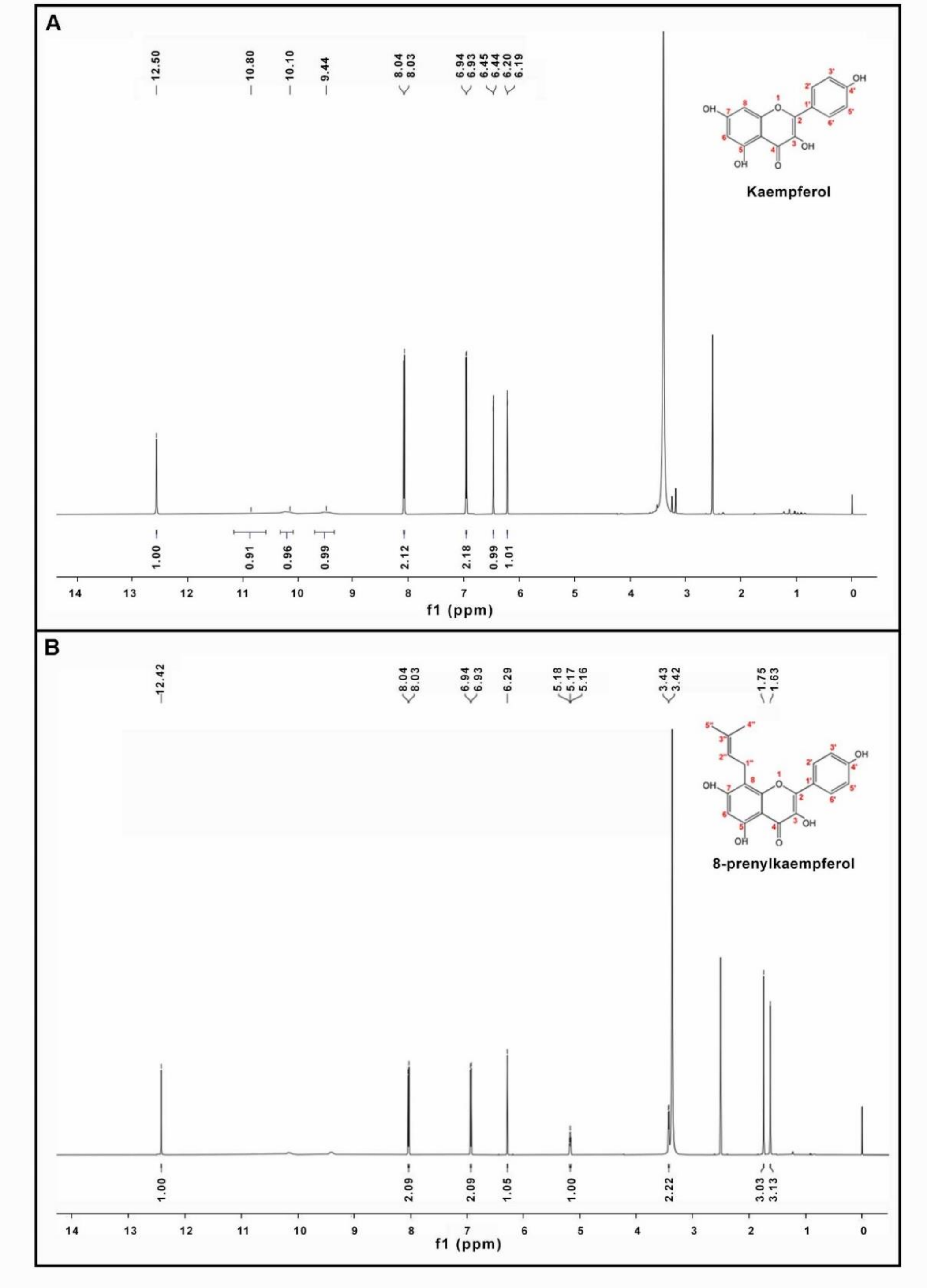

Figure S5. ^{1}H NMR spectrum of the enzymatic product of EpPT8 catalyzed reaction with kaempferol as substrate. (A) ^{1}H NMR (600 MHz) spectrum of kaempferol in DMSO-d_6. (B) 1H NMR (600 MHz) spectrum of 8-prenylkaempferol in DMSO-d6. The inserts illustrated their chemical structures, respectively.

2 Supplementary Tables

Table S1. Karyotype and genome features of five *Epimedium* species.

Taxa	Converted genome size (Gb/1C)	Chromosome number (2n)	Karyotype classification	Karyotype symmetry	Reference
Epimedium brevicornu Maxim.	3.14	12	4m (2sat) + 8sm	2A	(Zhang, 2018; Zhang et al., 2018)
Epimedium sagittatum (Sieb. Et Zucc.) Maxim	4.24	12	6m (2sat) + 6sm	2A	(Chen et al., 2012)
Epimedium pubescens Maxim.	4.43	12	6m (2sat) + 4sm+2st	2A	(Chen et al., 2012; Zhang et al., 2008)
Epimedium koreanum Nakai	4.10	12	6m (2sat) + 6sm	2A	(Zhang et al., 2018; Zhang et al., 2008)
Epimedium wushanense T. S. Ying	4.49	12	6m (2sat) + 6sm	2A	(Chen et al., 2012; Zhang et al., 2018)

Note: Karyotype classification includes m, median region; sm, submedian region; st, subterminal region; the original data of genome size unit was converted according to 1 pg = 978 Mb (Chen et al., 2012).

Table S2. The location record for wild populations of *E. pubescens* in China.

Record No.	Species	Location	Longitude	Latitude
1	*E. pubescens*	Baoxing county, yaan, Sichuan Province	102.82061	30.38835
2	*E. pubescens*	Yucheng district, Yaan, Sichuan Province	103.11986	29.58355
3	*E. pubescens*	Yucheng district, Yaan, Sichuan Province	103.03993	29.90817
4	*E. pubescens*	Yucheng district, Yaan, Sichuan Province	102.98368	30.23822
5	*E. pubescens*	Jianwei county, Leshan, Sichuan Province	103.93844	29.25571
6	*E. pubescens*	Shizhong district, GongLai, Sichuan Province	103.19144	30.26062
7	*E. pubescens*	Ermei Mountain, Sichuan Province	103.35633	29.57442
8	*E. pubescens*	Chongzhou, Sichuan Province	103.54000	30.82141
9	*E. pubescens*	Pengcheng, Sichuan Province	103.91928	31.19608
10	*E. pubescens*	Shuangliu district, Chendu, Sichuan Province	104.15686	30.30769
11	*E. pubescens*	Dujiangyan district, Chendu, Sichuan Province	103.36627	31.00452
12	*E. pubescens*	JinTang county, Chengdu, Sichuan Province	104.48548	30.76636
13	*E. pubescens*	Longquanze district, Chendu, Sichuan Province	104.16692	30.30853
14	*E. pubescens*	Hongya county, Meishan, Sichuan Province	103.10567	29.54398
15	*E. pubescens*	Qingshen country, Meishan, Sichuan Province	103.87124	29.77207
16	*E. pubescens*	Beichuan county, Mianyang, Sichuan Province	104.59590	31.93521
17	*E. pubescens*	Pingwu county, Mianyang, Sichuan Province	104.75149	32.41079
18	*E. pubescens*	Longchang county, Neijiang, Sichuan Province	104.64770	29.73899
19	*E. pubescens*	Hejiang county, Luzhou, Sichuan Province	105.83753	28.81850
20	*E. pubescens*	Anyue county, Ziyang, Sichuan Province	105.36226	30.11038
21	*E. pubescens*	Jiange county, Guangyuan, Sichuan Provicnce	105.28069	32.04828
22	*E. pubescens*	Cangxi county, Guangyuan, Sichuan Province	105.94140	31.73908
23	*E. pubescens*	Qingchuan county, Guangyuan, Sichuan Province	104.83319	32.52969
24	*E. pubescens*	Nanjiang county, Bazhong, Sichuan Province	106.48425	32.40037
25	*E. pubescens*	Bazhong county, Bazhong, Sichuan Province	106.75352	31.94683
26	*E. pubescens*	Enyang district, Bazhong, Sichuan Province	106.63709	31.79759
27	*E. pubescens*	Langzhong, Sichuan Province	106.44756	32.09486
28	*E. pubescens*	Dazu county, Chongqing	105.70735	29.55004
29	*E. pubescens*	Tongnan County, Chongqing	105.77277	29.85159
30	*E. pubescens*	Cheng County, Gansu Province	105.38433	33.43912
31	*E. pubescens*	Nanzheng district, Hanzhong, Shanxi Province	106.33866	32.46858
32	*E. pubescens*	Liuba county, Hanzhong, Shanxi Province	106.93690	33.61961
33	*E. pubescens*	Ningqiang county, Hanzhong, Shanxi Province	106.26286	32.83295
34	*E. pubescens*	Mian county, Hanzhong, Shanxi Province	106.67950	33.16002
35	*E. pubescens*	Lueyang county, Hanzhong, Shanxi Province	106.16325	33.33336
36	*E. pubescens*	Yang county, Hanzhong, Shanxi Province	107.55209	33.22917
37	*E. pubescens*	Taibai Mountain, Shanxi Province	107.82156	34.06204
38	*E. pubescens*	Shiquan county, Ankang, Shanxi Province	108.25452	33.04482
39	*E. pubescens*	Yang county, shangluo, Shanxi Province	109.70686	33.50044

Note: Wild populations were sampled at the field trips during 2018-2019.

Table S3. Sequencing data used for *E. pubescens* assembly.

Sequencing type	Number of reads	Average read length (bp)	Estimated coverage
Illumina paired end	5,521,463,927	150	230.94 ×
Oxford Nanopore	48,010,544	20,267	271.32 ×
Hi-C	1,965,567,758	150	94.88 ×

Note: Hi-C, High-through chromosome conformation capture.

Table S4. Summary of raw ONT data.

Raw ONT feature	Size (bp)	Number
Average length	20,267	N/A
N90	15,233	28,139,216
N80	19,643	22,552,532
N70	23,653	18,046,283
N60	27,725	14,249,100
N50	31,921	10,979,770
Longest	1,363,653	N/A
Total sequencing size	973,018,880,629	N/A
Total number (≥ 2 kb)	N/A	39,251,621

Note: ONT, Oxford Nanopore Technology; N/A, Not Available.

Table S5. Annotations of *E. pubescens* genome.

Database	Gene number	Percentage
GO	35,485	79.35%
KEGG	33,202	74.24%
KOG	25,592	57.22%
Pfam	35,782	80.01%
Swissprot	33,911	75.83%
TrEMBL	41,965	93.84%
eggNOG	36,370	81.32%
NR	42,016	93.95%
All Annotated	42,262	94.50%

Note: NR, NCBI non-redundant protein sequences; KEGG, Kyoto Encyclopedia of Genes and Genomes; KOG/COG/eggNOG, Clusters of Orthologous Groups of proteins; Pfam, Protein family; GO, Gene Ontology.

Table S6. Twelve plant species used in comparative genomic analyses.

Index	Order	Family name	Species name	Assembly accession number
1	Amborellales	Amborellaceae	*Amborella trichopoda*	GCF_000471905.2_AMTR1.0
2	Poales	Poaceae	*Oryza sativa*	GCF_001433935.1_IRGSP-1.0
3	Ranunculales	Ranunculaceae	*Aquilegia coerulea*	GCA_002738505_Aquilegia_coerulea_v1
4	Ranunculales	Ranunculaceae	*Coptis chinensis*	GCA_015680905.1_ ASM1568090v1
5	Ranunculales	Papaveraceae	*Papaver somniferum*	GCF_003573695.1_ASM357369v1
6	Ranunculales	Papaveraceae	*Macleaya cordata*	GCA_002174775.1_MC_HNAU_1.0
7	Brassicales	Brassicaceae	*Arabidopsis thaliana*	GCF_000001735.4_TAIR10.1
8	Vitales	Vitaceae	*Vitis vinifera*	GCF_000003745.3_12X
9	Gentianales	Rubiaceae	*Coffea arabica*	GCF_003713225.1_Cara_1.0
10	Solanales	Solanaceae	*Solanum lycopersicum*	GCF_000188115.4_SL3.0
11	Proteales	Nelumbonaceae	*Nelumbo nucifera*	GCF_000365185.1_Chinese_Lotus_1.1
12	Ranunculales	Berberidaceae	*E. pubescens*	The present study

Table S7. PCR primers for the amplification of different EpPT8 constructs.

Gene ID	Primer name	Sequence (5'-3')
EpPT8 (first round)	EpPT8Lf1	GTGAGGGAGAGACACAGAAC
	EpPT8Lr1	CAACATAACACGCAGTCTCTC
EpPT8 (second round)	EpPT8Lf2	AGCAGACAACAGCCATGGTT
	EpPT8Lr2	CAACATAACACGCAGTCTCTC
EpPT8	EpPT8Lf3	CACCATGGTTTCTAGATGTGCTTCT
	EpPT8Lr3	TTAACCAACGAATGGAATGAGT
*EpPT8*ΔTP82	EpPT8Lf4Δ82	CACCATGGCAACAAGTTCTGAAAAT
	EpPT8Lr4Δ82	TTAACCAACGAATGGAATGAGT
*EpPT8*ΔTP32	EpPT8Lf5Δ32	CACCATGAAACCAGGAACCAGAATAC
	EpPT8Lr5Δ32	TTAACCAACGAATGGAATGAGT

Note: *EpPT8*ΔTP82 and *EpPT8*ΔTP32, 82 and 32 amino acids truncated from the N terminus of *EpPT8*, respectively.

Table S8. Summary of assembled contigs from ONT data.

Assembly feature	Size (bp)	Number
N90	254,495	4,138
N80	389,708	3,022
N70	525,036	2,243
N60	692,316	1,657
N50	877,533	1,201
Longest	6,346,141	N/A
Total contigs	3,536,924,949	6,229

Table S9. Characteristics of pseudo-chromosomes by Hi-C.

Pseudo-chromosome	Anchored contigs	Anchored length (Mb)	Ordered contigs	Ordered length (Mb)	Gene number
Chr01	1,152	645.96	952	607.80	7,579
Chr02	1,194	615.34	1,001	586.90	7,611
Chr03	1,132	622.10	926	586.71	7,446
Chr04	1,002	575.86	844	548.07	7,477
Chr05	879	485.57	759	466.93	6,739
Chr06	817	460.22	680	437.45	5,975
Total	6,176	3,405.05	5,162	3,233.86	42,827

Table S10. Exon statistics from predicted gene models.

Exon number	Gene number	Percentage
Transcripts with 1 exon	9,149	20.46%
Transcripts with 2 exons	10,206	22.82%
Transcripts with 3 exons	6,467	14.46%
Transcripts with 4 exons	4,336	9.70%
Transcripts with 5 exons	3,197	7.14%
Transcripts with > 5 exons	11,367	25.42%
Total	44,722	

Table S11. Non-coding RNAs in *E. pubescens* genome.

Pseudo-chromosome	Rrna	miRNA	Trna	snoRNA	snRNA
Chr01	474	25	879	91	31
Chr02	386	37	1,583	51	43
Chr03	262	24	869	35	177
Chr04	105	38	716	40	29
Chr05	121	15	852	11	39
Chr06	132	25	572	38	111
Unassigned	233	8	323	12	28
Total	1,713	172	5,794	278	458

Table S12. Identification of repetitive elements in *E. pubescens* genome.

Type	Number	Length (bp)	Coverage
**Class I:Retroelement**	**3,550,242**	**2,245,994,178**	**62.61**
Class I/DIRS	1,460	219,668	0.01
Class I/LINE	227,819	87,492,563	2.44
Class I/LTR/Cassandra	99	5,942	1.65E-06
Class I/LTR/Caulimovirus	4,136	4,488,343	0.13
Class I/LTR/Copia	243,224	178,735,444	4.98
Class I/LTR/ERV	52,190	8,771,265	0.24
Class I/LTR/Gypsy	1,155,754	943,867,153	26.31
Class I/LTR/Pao	28,618	17,296,104	0.48
Class I/LTR/Viper	175	15,711	4.38E-06
Class I/SINE	19,576	2,965,262	0.08
Class I/LTR/Unknown	1,817,191	1,002,136,723	27.94
**Class II: DNA transposon**	**466,973**	**147,224,020**	**4.10**
Class II/Academ	194	11,748	3.27E-06
Class II/CACTA	78,364	28,900,259	0.81
Class II/Crypton	5,427	621,102	0.02
Class II/Dada	2,255	370,627	0.01
Class II/Ginger	2,460	237,447	0.01
Class II/Helitron	11,155	6,184,166	0.17
Class II/IS3EU	1,380	101,886	2.84E-05
Class II/Kolobok	5,470	1,199,699	0.03
Class II/MITE	1,033	125,957	3.51E-05
Class II/Maverick	2,291	163,850	4.56E-05
Class II/Merlin	426	24,575	6.84E-06
Class II/Mutator	23,547	16,615,931	0.46
Class II/Novosib	2,372	197,352	0.01
Class II/P	3,236	262,167	0.01

Continued

Type	Number	Length (bp)	Coverage
**Class II: DNA transposon**	583,950	276,608,409	7.92
Class II/PIF-Harbinger	24,903	8,916,941	0.25
Class II/PiggyBac	2,655	192,915	0.01
Class II/Sola	2,822	279,276	0.01
Class II/Stowaway	1	48	1.34E-08
Class II/Tc1-Mariner	33,428	10,804,977	0.30
Class II/Zator	217	15,880	4.42E-06
Class II/Zisupton	3,216	532,558	0.01
Class II/Hat	88,969	24,745,965	0.69
Class II/Unknown	171,152	46,718,694	1.30
**SSR**	14,863	8,183,433	0.23
**Total**	4,032,078	2,401,401,631	66.94

Table S13. LTR copia and LTR gypsy in 6 pseudo-chromosomes.

Pseudo-chromosome	Gypsy length (bp)	Percentage (%)	Copia length (bp)	Percentage (%)
Chr01	170,059,097	25.11	31,260,085	4.62
Chr02	163,092,995	25.28	33,060,954	5.12
Chr03	162,885,740	24.97	30,565,062	4.69
Chr04	152,844,065	25.31	28,881,589	4.78
Chr05	126,761,586	24.90	24,636,231	4.84
Chr06	120,064,765	24.88	24,645,457	5.11
Unassigned	48,158,905		5,686,066	
Total	943,867,153		178,735,444	
Ratio of total Gypsy and Copia		26.62		5.03

Table S14. BUSCO evaluation of *E. pubescens* genome.

BUSCO annotation	Number	Percentage
Complete BUSCOs	1,236	89.90%
Complete and single-copy BUSCOs	1,082	78.70%
Complete and duplicated BUSCOs	154	11.20%
Fragmented BUSCOs	47	3.40%
Missing BUSCOs	92	6.70%
Total BUSCO groups searched	1,375	100.00%

Note: BUSCO, Benchmarking universal single-copy orthologs.

Table S15. PTs of the UbiA superfamily used in the phylogenetic tree.

Protein name	Substrates	Plant species	Accession ID
OsPPT1	*p*-hydroxybenzoic acid	*Oryza sativa*	BAE96574.1
ZmPPT		*Zea mays*	NP_001148558.1
AtPPT1		*Arabidopsis thaliana*	NP_567688
LePGT-1		*Lithospermum erythrorhizon*	BAB84122.1
LePGT-2		*Lithospermum erythrorhizon*	BAB84123.1
GmVTE2-1	Homogentisate acid	*Glycine max*	ABB70126.1
TaVTE2-1		*Triticum aestivum*	ABB70123.1
ZmVTE2-1		*Zea mays*	ABB70122.1
CpVTE2-1		*Cuphea avigera*	ABB70125.1
AtVTE2-1		*Arabidopsis thaliana*	AAM10489.1
ApVTE2-1		*Allium ampeloprasum*	ABB70124.1
HvHGGT		*Hordeum vulgare*	AAP43911.1
OsHGGT		*Oryza sativa*	AAP43913.1
TaHGGT		*Triticum aestivum*	AAP43912.1
ZmHGGT		*Zea mays*	XP_008659772.1
GmVTE2-2		*Glycine max*	KRH71769.1
OsVTE2-2		*Oryza sativa*	XP_015646905.1
ZmVTE2-2		*Zea mays*	NP_001146703.1
DcVTE2-2		*Daucus carota*	XP_017246707.1
AtVTE2-2		*Arabidopsis thaliana*	ABB70127.1
AtrVTE2-2		*Amborella trichopoda*	XP_011628799.1
GmATG4	Chlorophyllide a/b	*Glycine max*	NP_001239633.1
OsATG4		*Oryza sativa*	ABO31092.1
ZmATG4		*Zea mays*	NP_001142204.1
AtATG4		*Arabidopsis thaliana*	NP_190750.1
GmCOX10	Haem B	*Glycine max*	XP_003556552.1
OsCOX10		*Oryza sativa*	EEC70799.1
AtCOX10		*Arabidopsis thaliana*	NP_566019.1

Continued

Protein name	Substrates	Species	Accession ID
AtABC4	1,4-dihydroxy-2-napthoic acid	*Arabidopsis thaliana*	NP_001117518.1
GmABC4		*Glycine max*	XP_003532605.1
OsABC4		*Oryza sativa*	NP_001049226.1
ZmABC4		*Zea mays*	NP_001152170.1
PcPT	Coumarin	*Petroselinum crispum*	BAO31627.1
PsPT1		*Pastinaca sativa*	AJW31563.1
PsPT2		*Pastinaca sativa*	AJW31564.1
ClPT1		*Citrus limon*	BAP27988.1
FcPT1a		*Ficus carica*	BBC82715.1
GmC4DT	Flavonoid	*Glycine max*	BAW32575.1
GmG2DT		*Glycine max*	BAW32578.1
GmG4DT		*Glycine max*	BAH22520.1
GmIDT1		*Glycine max*	BAW32576.1
GmIDT2		*Glycine max*	BAW32577.1
GmIDT3		*Glycine max*	XP_014618511.1
GmPT01		*Glycine max*	NP_001335591.1
GuA6DT		*Glycyrrhiza uralensis*	AIT11912.1
GuILDT		*Glycyrrhiza uralensis*	AMR58303.1
LaPT1		*Lupinus albus*	AER35706.1
LaPT2		*Lupinus albus*	AWK21939.1
LjG6DT		*Lotus japonicus*	ARV85585.1
PcM4DT		*Psoralea corylifolia*	AYV64464.1
SfFPT		*Sophora flavescens*	AHA36633.1
SfG6DT		*Sophora flavescens*	BAK52291.1
SfiLDT		*Sophora flavescens*	BAK52290.1
SfN8DT-1		*Sophora flavescens*	BAG12671.1
CsPT3		*Cannabis sativa*	DAC76713.1
CtIDT		*Cudrania tricuspidata*	AJD80983.1
MaIDT		*Morus alba*	AJD80982.1

Continued

**Protein name**	**Substrates**	**Species**	**Accession ID**
HlPT-1	Phloroglucinol	*Humulus lupulus*	BAJ61049.1
HlPT2		*Humulus lupulus*	AJD80255.1

Note: PTs, prenyltransferases; UbiA, ubiquinone biosynthesis gene.

Table S16. Comparison of enzymatic activity between EsPT2 and EpPT8

Substrate names	Conversion rate (%)	
	EpPT8	EsPT2
Kaempferol	71.02%	21.88%
Quercetin	34.81%	15.65%

Note: Conversion rate was calculated as: peak area of reaction product/peak area of the sum of substrate and product.
