# Supplementary figures and images for: The discovery of a key prenyltransferase gene assisted by a chromosome-level Epimedium pubescens genome
Source: Front Plant Sci. 2022 Nov 14;13:1034943. doi: 10.3389/fpls.2022.1034943 (PMC9702526; doi:10.3389/fpls.2022.1034943)

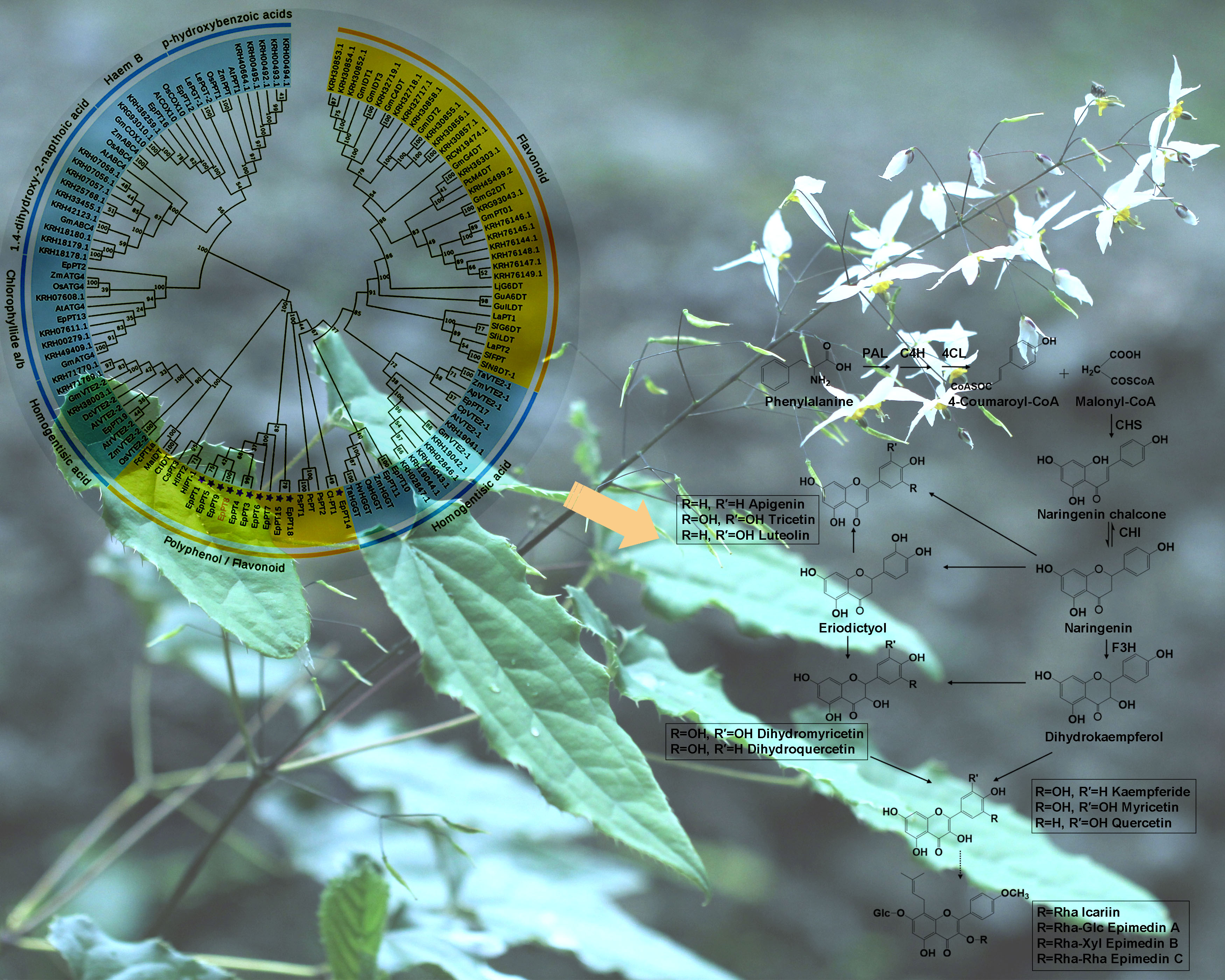

Supplement: Supplementary file 2 [file Image_1.jpeg]
